# Growing hulled wheat and old bread wheat genotypes in non-marginal environments: agronomic, qualitative, and safety insights

**DOI:** 10.3389/fpls.2025.1638426

**Published:** 2025-11-06

**Authors:** Claudia Sardella, Francesca Vanara, Mattia Scapino, Valentina Scarpino, Clara Pedrazzani, Chiara Dall’Asta, Massimo Blandino

**Affiliations:** 1 Department of Agricultural, Forest and Food Sciences, University of Turin, Turin, Italy; 2 Department of Food and Drug, University of Parma, Parma, Italy

**Keywords:** yield, grain quality, alkylresorcinols, phenolic acids, rheology, deoxynivalenol, gliadins

## Abstract

Hulled wheats (einkorn, emmer, and spelt) and old genotypes of bread wheat (*T. aestivum* L. ssp. *aestivum*) have been attracting attention in plant breeding and in the food industry for food diversification and value addition purposes. This study investigated the agronomic behavior, the grain quality, the contaminants, the bioactive compounds (e.g., alkylresorcinols and phenolic acids), and the antioxidant capacity (FRAP and DPPH) of modern genotypes of hulled wheat (einkorn, emmer, and spelt), as well as old Italian bread wheat genotypes, under standard cropping conditions. These genotypes were compared with modern bread wheat genotypes used as commercial references in four location-by-year experiments in Northwest Italy. The treatments were assigned to experimental units using a completely randomized block design and three replications. The modern bread wheat genotypes displayed better agronomic performance, while the old bread wheat genotypes had higher protein contents, but were characterized by higher dough weakening, even with increasing levels of nitrogen fertilization. The old genotypes did not offer advantages in terms of toxic celiac disease epitopes, but exhibited an overall reduced susceptibility to mycotoxin accumulation, although a large variability was observed within the modern hulled and bread wheat genotypes. The bioactive compound content and the antioxidant capacity were closely related to the growing conditions. Majority of the differences among the genotypes emerged from the soluble fraction of phenolic acids, with a slightly higher content in the old bread wheat genotypes than in the modern ones and a different profile for the hulled wheats. On the other hand, the alkylresorcinol content was higher in the modern genotypes than in the old ones and was closely related to kernel size. Overall, the results highlighted considerable differences among genotypes and a broader variability in the modern genotypes than the old ones for many of the studied traits. Substantial agronomic and qualitative differences were observed within the hulled wheats, thus suggesting opportunities for genetic selection and the development of improved hulled or bread wheat genotypes.

## 1 Introduction

Wheat (*Triticum* spp.) has been a primary component of the human diet since its first cultivation, approximately 10,000 years ago (Salamini et al., 2002). The earliest domesticated species included einkorn (*T. monococcum* ssp. *monococcum*, genome AA), emmer (*T. turgidum* ssp. *dicoccum*, genome AABB), and, later, spelt (*T. aestivum* ssp. *spelta*, genome AABBDD). These species are characterized by hulled grains, in which the glumes remain tightly adherent to the grain and are not easily released by threshing. Bread wheat (BW), also known as common wheat (*T. aestivum* ssp. *aestivum*, genome AABBDD), originated approximately 9,000 years ago through hybridization between cultivated emmer *T. tauschii* and has existed exclusively under cultivation (Arzani and Ashraf, 2017). The domestication of BW involved the fixation of several key traits, such as the transition from brittle to non-brittle rachis, reduction of spike shattering at maturity, and from hulled to free-threshing forms, in which grains are easily released from the glumes upon threshing. BW and spelt belong to the same hexaploid species; however, BW benefited from its free-threshing characteristic, which sets it apart from the earliest cultivated wheats (Shewry, 2018). Today, BW is the most widely cultivated wheat type, accounting for around 95% of global production. Durum wheat (*T. turgidum* ssp. *durum*, genome AABB) is awned, meaning it has bristles, and it accounts for the remaining 5% (FAOSTAT, 2023). Einkorn, emmer, and spelt are often referred to as hulled or ancient wheats. These terms denote early domesticated hulled species that represented a bridge between their wild ancestors and the cultivated, free-threshing wheat types (bread and durum wheat), which replaced them over time. In contrast, old genotypes of bread and durum wheat, also known as ancient or heirloom genotypes, refer to varieties that have been cultivated for a long time and not significantly modified through modern breeding practices. These include autochthonous, regionally adapted BW genotypes that were gradually replaced from the 1960s onward by the semi-dwarf, high-yielding modern genotypes developed through systematic breeding (Boukid et al., 2018; Mefleh et al., 2022). These genotypes offer unique characteristics and can be valuable resources for wheat breeding and food production.

Despite their limited commercial relevance, hulled wheat varieties are still cultivated in small areas, particularly in marginal environments, for niche or high-value markets. They have not been subjected to the extensive selection and genetic improvement typical of modern breeding programs targeting bread and durum wheat. However, in recent decades, breeding efforts have also targeted hulled wheats, particularly spelt. This has led to the development and the release of modern genotypes of einkorn, emmer, and spelt after the year 2000 (Longin et al., 2016). These new lines exhibit improved agronomic traits, including a reduced plant height, an increased harvest index, and an enhanced yield potential, although they remain distinct from the modern genotypes in terms of domestication history. Parallel to this, a renewed interest in old genotypes has also emerged. Both the hulled wheat species and the old genotypes of BW are often perceived as safer, healthier, and more environmentally sustainable than the modern ones (Guarda et al., 2004; Shewry, 2018). Their derived products, which are often marketed as artisanal and specialty foods, align with the growing demand among consumers for value-added options. Moreover, despite their relatively low yield potential, traits such as their relatively low nutrient requirements and their adaptability to marginal conditions have made them highly attractive crop options, especially for organic farming (Migliorini et al., 2016). Extensive research (Di Silvestro et al., 2012; Dinelli et al., 2011; Ziegler et al., 2016) has been carried out on both hulled wheat and old BW genotypes to explore the perceived benefits that promoted their reintroduction, emphasizing their good phytochemical compound contents under limited input conditions and their potential to enhance the nutritional value of bread formulations. However, direct comparisons with modern BW genotypes are limited, particularly for non-marginal production systems (Shewry and Hey, 2015).

Furthermore, some concerns have emerged regarding the impact of breeding on the nutritional traits. The selection for increased grain size and starch accumulation in modern BW may have inadvertently reduced the concentration of essential minerals, such as iron and zinc, as well as proteins, due to a yield dilution effect, as these components do not scale proportionally with the grain biomass (Simsek et al., 2019; Fan et al., 2008; Shewry and Hey, 2015). However, the lower grain protein concentration has been matched by a quality improvement in the protein composition over the last few decades, with greater strength values of the flour, which have better suited the technological needs of the market (Guarda et al., 2004; Ormoli et al., 2015; Singh et al., 2021). The selection of high gluten strength, which has acted more on the glutenin genes, thus decreasing the gliadin/glutenin ratio, supports the conclusions of some authors (Ribeiro et al., 2016) who have observed a reduction in the number of toxic gliadin epitopes for patients with celiac disease in modern BW genotypes compared with the old ones. Breeding efforts over the last century were also aimed at increasing disease resistance to prevent yield and quality losses and to limit the health-related risks posed by contamination of the grains by toxic fungal metabolites, such as the mycotoxins produced by *Fusarium* head blight (FHB) pathogens. In the past few decades, knowledge of genetic variations related to nutritional and dough quality traits, as well as pathogen resistance, has increased, and landraces, wild relatives, and hulled wheat lines have been actively screened and used as sources of genetic diversity in modern wheat breeding (Buerstmayr et al., 2020; Singh et al., 2025).

Despite this growing body of knowledge, the effective reintegration of hulled wheat and old BW genotypes into modern food systems requires a better understanding of their performance under conventional agronomic conditions, particularly in comparison with modern BW genotypes. Hulled wheat and old BW genotypes have generally been compared with reference material under subsistence and low-input conditions, while modern genotypes are typically bred for high-input production systems. The ongoing demand of the market and, in particular, of the industrial supply chain points out the need for an expansion of the cultivation of these genotypes, even in non-marginal cereal-growing areas. This motivated us to investigate the agronomic performance and the grain quality of modern genotypes of einkorn, emmer, and spelt and old Italian genotypes of BW in a series of field trials under state-of-the-art wheat production and conventional agronomic practices. These genotypes were compared with modern, reference BW genotypes for the Italian market, which are characterized by diverse technological properties and commercial uses. Several key agronomic, composition, and qualitative traits, including the grain phytochemical composition and the mycotoxin accumulation, were investigated to fully evaluate the behavior of the genotypes in the considered production situations and to assess their potential role in food systems. In addition, a representative subset of old and modern BW genotypes was studied under three varying input rates of nitrogen (N) fertilization, a commonly applied agronomic practice that could strongly affect both the grain yield (GY) and the quality for bread-making. This information was used to draw conclusions about optimized farming and processing and to more appropriately guide the agronomic choices of farmers and food supply chain operators.

## 2 Materials and methods

### 2.1 Experimental design

A total of 13 *Triticum* spp. genotypes, including diploid, tetraploid, and hexaploid species (**Supplementary Table S1**), were considered for the experiment. The einkorn, emmer, and spelt were selected from among improved genotypes released after 2000. The old genotypes of BW were chosen from among the landraces cultivated in the 19th century and from the old genotypes developed in the 20th century from varietal crosses, all released before 1953 (Migliorini et al., 2016). The selected modern BW genotypes, used as a control, were released after 2000 and were chosen as references, in the Italian context, of three different quality categories—wheat for biscuits (FB, frumento biscottiero), for ordinary bread-making (FP, frumento panificabile), and improver high protein (FF, frumento di forza)—on the basis of their potential technological use according to the Synthetic Index of Quality (Foca et al., 2007). The studied genotypes were cultivated over two growing seasons (2016–2017 and 2017–2018) in two different locations in Northwest Italy: a field in Carmagnola (44° 50′ N, 7° 40′ E, 245 m a.s.l.), in a deep fertile loam-silty soil, and a field in Cigliano (45° 18′ N, 8° 01′ E, 237 m a.s.l.), in a shallow sandy–loam soil, using the same experimental setup.

The treatments were assigned to experimental units using a completely randomized block design, with a 10.5-m^2^ plot (7 m × 1.5 m), and three replications.

**Supplementary Table S2** details the physical and chemical characteristics of the soils, which were sampled as described by Sardella et al. (2023a) just before the N fertilization at tillering, i.e., growth stage (GS) 23, according to the BBCH (Biologische Bundesanstalt, Bundessortenamt und CHemical Industry) scale (Zadoks et al., 1974). Precipitation and daily temperature data were collected from meteorological stations located in the Carmagnola experimental farm and 3 km away from the Cigliano experimental field. Cumulative rainfall and growing degree days (GDDs) were calculated for each month and are reported in **Supplementary Table S3**.

The agronomical practices that are standard for the area and for the crops under comparison were adopted using a conventional management system. Briefly, in both years, BW and maize were the preceding crops in Carmagnola and Cigliano, respectively, and the fields were ploughed (30 cm) and disk harrowed each year. After plowing and before harrowing, 50 kg ha^−1^ P_2_O_5_ (as granular triple phosphate) and 66 kg ha^−1^ K_2_O (as granular potassium chloride) were applied. Planting was performed at the end of October in 12-cm-wide rows at a seeding rate of 300 seeds/m^2^ for einkorn, emmer, and spelt and 400 seeds/m^2^ for BW. The N fertilizer (80 kg N ha^−1^) was manually provided to all the plots as ammonium nitrate (granular, N 27%) and equally split into two dosages at the tillering stage (GS 23) and at the beginning of stem elongation (GS 32). Weed control was conducted chemically at GS 23 with pinoxaden and cloquintocet-mexyl (Axial Pronto^®^, Syngenta Italia, Milan, Italy) and fluroxipir, clopiralid, and MCPA (Manta Gold^®^, Syngenta Italia) at the manufacturers’ recommended field rates. This was performed using a tractor-mounted boom sprayer equipped with T-Jet AI110/04 nozzles. No fungicides or insecticides were used. **Supplementary Table S4** details the timing of the main agronomic management practices applied to the experimental plots.

In addition to the comparison between genotypes in different environmental conditions, a specific agronomic and qualitative study was carried out on BW in the sandy-loam soil. Only in the Cigliano experimental field (2016–2017 and 2017–2018) were three N fertilization treatments applied, as ammonium nitrate (N_0_, N_80_, and N_160_, at fertilization rates of 0, 80, and 160 kg N ha^−1^, respectively), to the old and modern BW genotypes in order to evaluate the effects of varying N levels on the agronomic and rheological parameters. The total amount of administered N was equally split into two dosages between the tillering stage (GS 23) and the beginning of stem elongation (GS 32) to avoid leaching and to improve N availability, according to the common N fertilization practices in the growing areas.

### 2.2 Agronomic and physical parameters of the grains

A few crop indices were determined during the growth cycle. The area under the canopy greenness curve (AUCGC), which expresses both the vigor and the chlorophyll content of a plant, was calculated throughout the growing seasons according to Capo and Blandino (2021). The ear density (number of ears per unit of grown area), the average plant height, and the percentage of plot with lodging were recorded for each plot (Sardella et al., 2023b). Harvesting was carried out using a Walter Wintersteiger cereal plot combine harvester at the beginning of July (**Supplementary Table S4**), and the straw was collected behind the combine harvester and weighed immediately after harvesting. The GY and the straw yield were calculated on a plot basis and adjusted to a 13% moisture content, while the harvest index (HI) values were expressed as a percentage (Sardella et al., 2023b). After harvesting, the einkorn, emmer, and spelt husks were removed using a laboratory dehusking machine (FC2K Otake, Dellavalle S.r.l., Mezzomerico, Italy). The harvested grains were thoroughly mixed, and 2 kg samples were taken from each plot for the determination of moisture content, test weight (TW), and thousand-kernel weight (TKW), as described by Sardella et al. (2023a).

### 2.3 Qualitative parameters

Representative subsamples (500 g) were ground to wholemeal (Sardella et al., 2023a) to determine the grain protein content (GPC) and the ash content according to the AACC method 39-10.01 (AACC International, 2008). For chemical analyses, the samples were ground to a fine powder (particle size, <500 μm) and stored at −25 °C.

### 2.4 Rheological parameters

Only the grains of the old and modern BW genotypes from the Cigliano experimental field, which were treated with different N fertilization rates, were ground to refined flour to examine the mixing and pasting properties. A 2-kg grain sample was taken from each plot and milled using the Bona 4RB mill (Bona, Monza, Italy) to provide representative subsamples of refined flour. The analyses were performed using a fast Mixolab^®^ (Chopin Technologies, Villeneuve-la-Garenne, France) mixing protocol, according to the ICC standard method 173 (ICC, 2011), with 75 g of flour and a constant water absorption (WA) of 55%, as described by Sardella et al. (2023b). This instrument allows obtaining specific information about the behavior of dough constituents (starch, protein, and water) by continuously measuring the torque (in Newton-meters, Nm) produced by the passage of the dough between two mixing blades submitted to both shear stress and temperature changes (Banu et al., 2011).

### 2.5 Gliadin quantification by R5 ELISA

The refined flour of the considered genotypes was analyzed with RIDASCREEN^®^ Gliadin (art. no. R7001; R-Biopharm AG, Darmstadt, Germany) to determine the gliadin content (mg kg^-1^) as a measure of the gluten in food. This test involves conducting a sandwich enzyme-linked immunoassay (ELISA) based on a specific monoclonal antibody, i.e., R5, which is able to recognize toxic celiac sequences such as QQPFP, QLPFP, LQPFP, and QQQFP (Van Eckert et al., 2010). The samples were extracted with Cocktail Solution (art. no. R7006/R7016, patent WO 02/092633; R-Biopharm AG), which was used according to the manufacturer’s instructions.

### 2.6 Deoxynivalenol analysis

The harvested grains were mixed thoroughly, and 4 kg grain samples were taken from each plot and ground completely using a Retsch ZM 200 (Retsch GmbH, Haan, Germany) fitted with a 1-mm aperture sieve to provide a homogenous particle dimension. A representative subsample of the milled material was analyzed for deoxynivalenol (DON) content using the ELISA method (Nguyen et al., 2019) with RIDASCREEN^®^ DON direct competitive immunoassays (R-Biopharm AG), in accordance with the manufacturer’s instructions. Briefly, mycotoxins were extracted for 15 min by mechanical shaking at 100 rpm (MOM Instruments, Milan, Italy) of 20 g samples with 100 mL of distilled water. After extraction and filtration through Whatman^®^ no. 1 filters, 50 µL of diluted filtrate was used for the ELISA test. The optical density was measured at 450 nm using an ELISA 96-well plate reader (Das S.r.l., Rome, Italy), and all the standard and sample solutions were analyzed in duplicate wells. The limit of detection (LOD) of the analytical method was set at 37 µg kg^−1^.

### 2.7 Phytochemical compound analyses

#### 2.7.1 Chemicals

The used 2,2-diphenyl-1-picrylhsydrazyl (DPPH), ethanol (CHROMASOLV^®^, 99.8%), ethyl acetate (CHROMASOLV^®^, 99.8%), (±)-6-hydroxy-2,5,7,8-tetramethylchromane-2-carboxylic acid (Trolox, 97%), hydrochloric acid (37%), iron(III) chloride hexahydrate (FeCl_3_·6H_2_O, ≥98%), methanol (CHROMASOLV^®^, 99.9%), sodium hydroxide (≥98%), 2,4,6-tris(2-pyridyl)-*s*-triazine, butylated hydroxytoluene (≥99%), phenolic acid standards (caffeic acid ≥98%, *p*-coumaric acid ≥98%, ferulic acid ≥99%, gallic acid ≥99%, protocatechuic acid ≥99%, *p*-hydroxybenzoic acid ≥99%, sinapic acid ≥98%, syringic acid ≥95%, and vanillic acid ≥97%), and alkyl resorcinol standards (5-nonadecyl-resorcinol, 5-heneicosylresorcinol, 5-tricosyl-resorcinol, and 5-heptadecylresorcinol, all 10 mg powder) were purchased from Sigma-Aldrich (St. Louis, MO, USA). Water (HPLC grade) was obtained from ELGA PURELAB Ultra system (M-Medical, Cornaredo, Milan, Italy). LC-MS grade methanol, ethyl acetate, and 2-propanol were purchased from Scharlab Italia S.r.l. (Milan, Italy). MS-grade ammonium formate and formic acid were obtained from Fisher Chemical (Thermo Fisher Scientific, Inc., San Jose, CA, USA).

#### 2.7.2 Extraction and quantification of the phenolic acids and the total alkylresorcinol content

The soluble (free and conjugated, SPAs) and cell wall-bound (CWBPAs) phenolic acids were extracted according to the procedure reported in Giordano et al. (2019). Briefly, the SPAs were extracted from a 0.1250-g sample twice with 1 mL of an 80:20 (*v*/*v*) ethanol/water solution in an ultrasound bath (Bandelin Electronic, Berlin, Germany) at 4 °C for 10 min. The supernatants were collected and then evaporated to dryness under a nitrogen stream. The samples were hydrolyzed with 2 M NaOH (400 μL) under continuous stirring at 4 °C for 2 h. After acidification to pH 2 with HCl 6 N (160 μL), the SPAs were extracted three times with 500 μL of ethyl acetate. The combined supernatants were evaporated to dryness under a nitrogen stream and then reconstituted in 100 μL of an 80:20 (*v*/*v*) methanol/water solution. The previously described extraction was repeated for the CWBPAs, and the supernants were discarded. Subsequently, hydrolysis was performed by adding 400 mL of 2 M NaOH to the remaining pellet. The mixture was stirred continuously at 4 °C for 4 h. After acidification to pH 2 with HCl 12 N (120 μL), the CWBPAs were extracted with 800 μL of ethyl acetate and then centrifuged at 10,600 × *g* for 2 min at 4 °C. The extraction was repeated once more. The combined supernatants were evaporated to dryness under a nitrogen stream and then reconstituted in 200 μL of an 80:20 (*v*/*v*) methanol/water solution. Both the SPA and CWBPA extracts were filtered through a 0.2-μm filter and then analyzed using a high-performance liquid chromatography Agilent 1200 Series (Agilent Technologies, Santa Clara, CA, USA) coupled to an Agilent 1200 Series diode array detector, as described in Giordano et al. (2019).

The alkylresorcinols (ARs) were extracted following Pedrazzani et al. (2021) and were analyzed according to Righetti et al. (2019). Briefly, 1 g of sample was stirred for 60 min at 240 strokes per minute with 20 mL of ethyl acetate, and then the mixture was centrifuged for 10 min at 14,000 rpm. The resulting supernatant (1,000 μL) was dried under a nitrogen flow. After two repetitions, the supernatants were pooled, reconstituted with 1 mL of mobile phase B, and injected into an ultrahigh-performance liquid chromatography (UHPLC)/travelling-wave ion mobility spectrometry (TWIMS)–quadruple time-of-flight (QTOF) system. AR quantification was based on external standard calibration (range, 0.1–25 mg kg^−1^), which was performed based on Pedrazzani et al. (2021). The results for both phenolic acids and ARs were expressed as mg kg^−1^ of dry weight (DW).

#### 2.7.3 Antioxidant capacity determination

The total antioxidant capacity (AC) was measured directly, using the QUENCHER procedure, on the wholemeal flour samples by means of two assays and expressed as mmol Trolox equivalents (TE) kg^−1^ of DW. Both the DPPH scavenging capacity (AC_DPPH_) and the ferric reducing antioxidant power (AC_FRAP_) were determined according to Serpen et al. (2012).

### 2.8 Statistical analyses

Given the marked differences in the soil characteristics and the weather conditions, four different experiments were set up: SL17 (Cigliano, 2016–2017, sandy-loam soil), LS17 (Carmagnola, 2016–2017, loam-silty soil), SL18 (Cigliano, 2017–2018, sandy-loam soil), and LS18 (Carmagnola, 2017–2018, loam-silty soil). The data obtained from the plots supplied with 80 kg N ha^−1^ allowed a comparison to be made of all the genotypes across species in the four experiments. Three biological replications were considered for each treatment. The data were assessed for normality and homoscedasticity. Analysis of variance (ANOVA) was performed using the SPSS statistical package, version 29.0.1.0 (SPSS Inc., Chicago, IL, USA). The genotype (G; *n* = 13) and the experiment (E; *n* = 4) were considered as fixed effects. When single factors, or their interaction, determined a significant effect, the means were compared using the Ryan–Einot–Gabriel–Welsch *F* (REGW-*F*) *post-hoc* test, with *p*-value < 0.05. Simple correlation coefficients were obtained for the phytochemical compounds and the AC.

The effect of N fertilization on the agronomic and rheological parameters was examined using ANOVA, considering only the old and modern BW genotypes cultivated in the sandy-loam soil at Cigliano over the 2 years. The treatments were factorial combinations of eight genotypes (G), three N fertilization rates (N), and two harvest years (Y), which were considered as fixed effects. Means were identified as significantly different on the basis of the REGW-*F* statistical test.

Broad-sense heritability (*H*^2^) was estimated using linear mixed models implemented through the H2cal function from the inti package in R (R Core Team, 2020). For each trait, a mixed-effects model was fitted with the following structure:

- Fixed model: 0 + (1|block) + (1|E) + G
- Random model: 1 + (1|G) + (1|block) + (1|E) + (1|G:E) + (1|G:Year) + (1|G:E:Year)

Genotype and its interactions with environment and year were treated as random effects, while block and environment were included as fixed effects.

Principal component analysis (PCA) was performed to summarize the relationship among the agronomic and qualitative parameters and the compared genotypes using the free statistical software R version 4.2.2 (R Core Team, 2020). Data were preliminary standardized by subtracting the means and dividing by the standard deviations within each variable.

## 3 Results

### 3.1 Meteorological data

The meteorological data showed remarkable differences in the monthly cumulative rainfall and the GDDs across years, particularly in April and May (**Supplementary Table S3**). An excess rainfall level in the 2017–2018 period from booting (April) to flowering (May) favored a higher FHB infection, especially in the Carmagnola site (LS18), with a strong impact on the agronomic and qualitative parameters.

### 3.2 Agronomic and grain qualitative parameters

**Figure 1:**
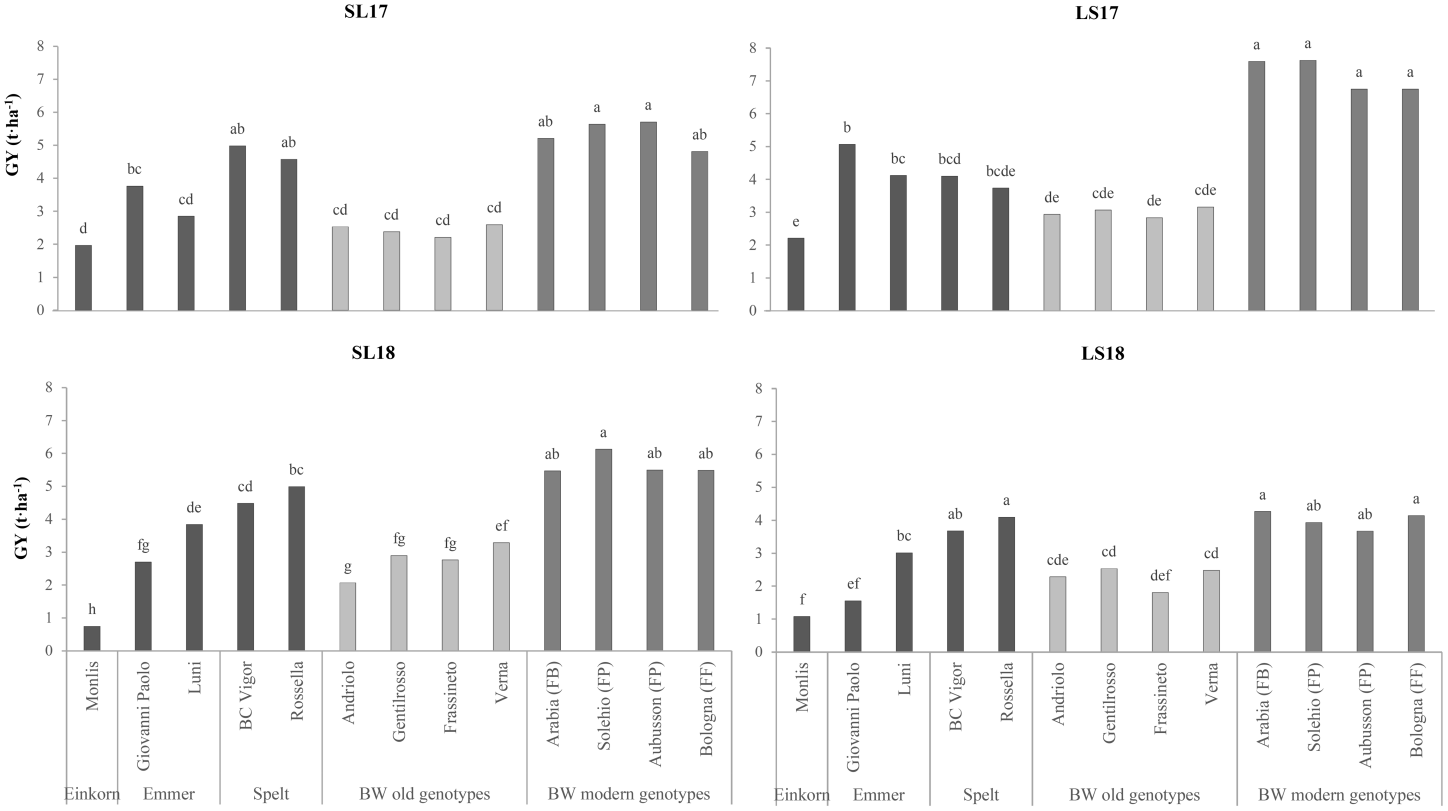
Comparison of the grain yields (GY) of the investigated genotypes across the growing environments. *SL17*, sandy-loam soil, harvested in 2017; *LS17*, loam-silty soil, harvested in 2017; *SL18*, sandy-loam soil, harvested in 2018; *LS18*, loam-silty soil, harvested in 2018; *BW*, bread wheat; *FB*, wheat for biscuits (frumento biscottiero); *FP*, ordinary bread-making wheat (frumento panificabile); *FF*, improver high-protein wheat (frumento di forza). Different letters above the columns indicate significant differences between the six groups for *p* (*F*) < 0.05 according to the REGW-*F* test.

Two-way ANOVA, carried out on the agronomic and qualitative data, revealed highly significant differences for the genotype and the experiment factors (**Table 1**). The GY differentiated the genotypes according to the species, with the modern ones presenting the highest values (mean value of 5.5 t ha^−1^) and significantly lower yields for spelt (−23%), emmer (−42%), the old wheat genotypes (−51%), and einkorn (−68%). Significant differences were found between experiments, as the plots grown in LS17 performed better (4.7 t ha^−1^) than those in SL17 and SL18 (−21%), while LS18 showed the lowest average yield (−36%, compared with the highest-yielding experiment) despite the good fertility of the soil. The genotypes responded differently to the improved yield conditions (**Figure 1**), and the modern BW genotypes in particular, which showed an improved responsiveness to better growing conditions. The old BW genotypes were the least productive of all the considered production systems, while the spelt genotypes performed better for a low nutrient availability of the soils (+20% in both SL17 and SL18 compared with LS17).

**Table 1:** Effect of the genotype, the experiment, and their interaction on the agronomic traits and qualitative parameters of the investigated genotypes cultivated in the experimental trials and supplied with 80 kg N ha^−1^.

Factor	Crop		Source of variation	GY (t ha^−1^)		Lodging (%)		Ear density (n° m^−2^)		TKW (g)		TW (kg hL^−1^)		GPC (%)		Ash (%)	
Genotype (G)	Einkorn		Monlis	1.5	h	37.0	cd	741	a	28.4	h	71.7	de	16.1	a	2.17	a
	Emmer		Giovanni Paolo	3.4	de	6.7	e	335	e	49.7	b	63.6	f	16.1	ab	1.92	d
			Luni	3.5	d	45.3	bc	474	bcd	46.1	c	61.2	g	13.7	e	1.90	def
	Spelt		BC Vigor	4.3	c	26.0	cde	459	bcd	48.9	b	64.2	f	15.6	abcd	2.02	bc
			Rossella	4.5	c	14.0	de	434	d	53.5	a	64.4	f	12.6	f	1.87	f
	BW	Old genotypes	Andriolo	2.5	fg	86.9	a	530	bcd	43.9	de	74.4	bc	15.7	abc	2.05	b
			Gentilrosso	2.7	fg	84.7	a	536	bc	50.1	b	75.0	b	15.0	cd	2.00	c
			Frassineto	2.4	g	80.3	a	493	bcd	49.8	b	74.5	bc	14.9	d	2.01	c
			Verna	2.9	ef	67.1	ab	535	bc	42.7	e	75.5	b	15.3	bcd	2.05	b
		Modern genotypes	Arabia (FB)	5.7	ab	6.7	e	443	cd	42.8	e	73.2	cd	12.7	f	1.88	def
			Solehio (FP)	5.8	a	14.0	de	492	bcd	45.0	cd	74.0	bc	12.1	f	1.91	de
			Aubusson (FP)	5.4	ab	14.4	de	530	bcd	36.8	f	70.3	e	12.6	f	1.86	f
			Bologna (FF)	5.3	b	7.0	de	538	b	32.9	g	77.2	a	13.5	e	1.88	ef
			*p* (F)	***		***		***		***		***		***		***	
Experiment (E)			SL17	3.8	b	32.0	bc	442	c	48.3	a	73.8	a	12.6	b	1.87	c
			LS17	4.7	a	59.6	a	614	a	44.3	b	71.1	c	15.8	a	1.97	b
			SL18	3.7	b	21.0	c	325	d	44.9	b	72.0	b	12.8	b	1.98	b
			LS18	3.0	c	42.4	b	561	b	37.9	c	66.8	d	16.0	a	2.04	a
			*p* (F)	***		***		***		***		***		***		***	
G × E			*p* (F)	***		ns		ns		***		***		***		***	
*H* ^2^				0.98		0.96		0.88		0.99		0.99		0.98		0.98	

Means followed by different letters are significantly different, according to the REGW-*F* test [(*) *p* (F) < 0.05, (**) *p* (F) < 0.01, (***) *p* (F) < 0.001, and ns, non-significant]. Broad-sense heritability (*H*^2^) was estimated across genotypes and experiments.

*GY*, grain yield; *TKW*, thousand kernel weight; *TW*, test weight; *GPC*, grain protein content; *BW*, bread wheat; *FB*, wheat for biscuits (frumento biscottiero); *FP*, ordinary bread-making wheat (frumento panificabile); *FF*, improver high-protein wheat (frumento di forza); *SL17*, sandy-loam soil, harvested in 2017; *LS17*, loam-silty soil, harvested in 2017; *SL18*, sandy-loam soil, harvested in 2018; *LS18*, loam-silty soil, harvested in 2018.

The highest lodging percentage was recorded for the old BW genotypes (mean value of 80%). The response of the hulled wheats differed according to the specific genotypes, mainly related to the plant height. The old BW genotypes were, on average, 60 cm taller than the modern BW genotypes, while the hulled wheat genotypes, such as the spelt Rossella and the emmer Giovanni Paolo, were characterized by lower heights and were also less prone to lodging (<14%), similarly to the modern BW genotypes. As expected, large differences in HI were observed for modern BW (around 50%) compared with the hulled and old BW genotypes (HI < 40%, with the emmer Monlis presenting an extremely low value of around 15%), except for the spelt Rossella (around 50%). Lodging was affected significantly by the experiment factor, with higher values observed in the experiment characterized by high fertility of the soil (LS17, in particular for the old BW genotypes).

**Figure 2:**
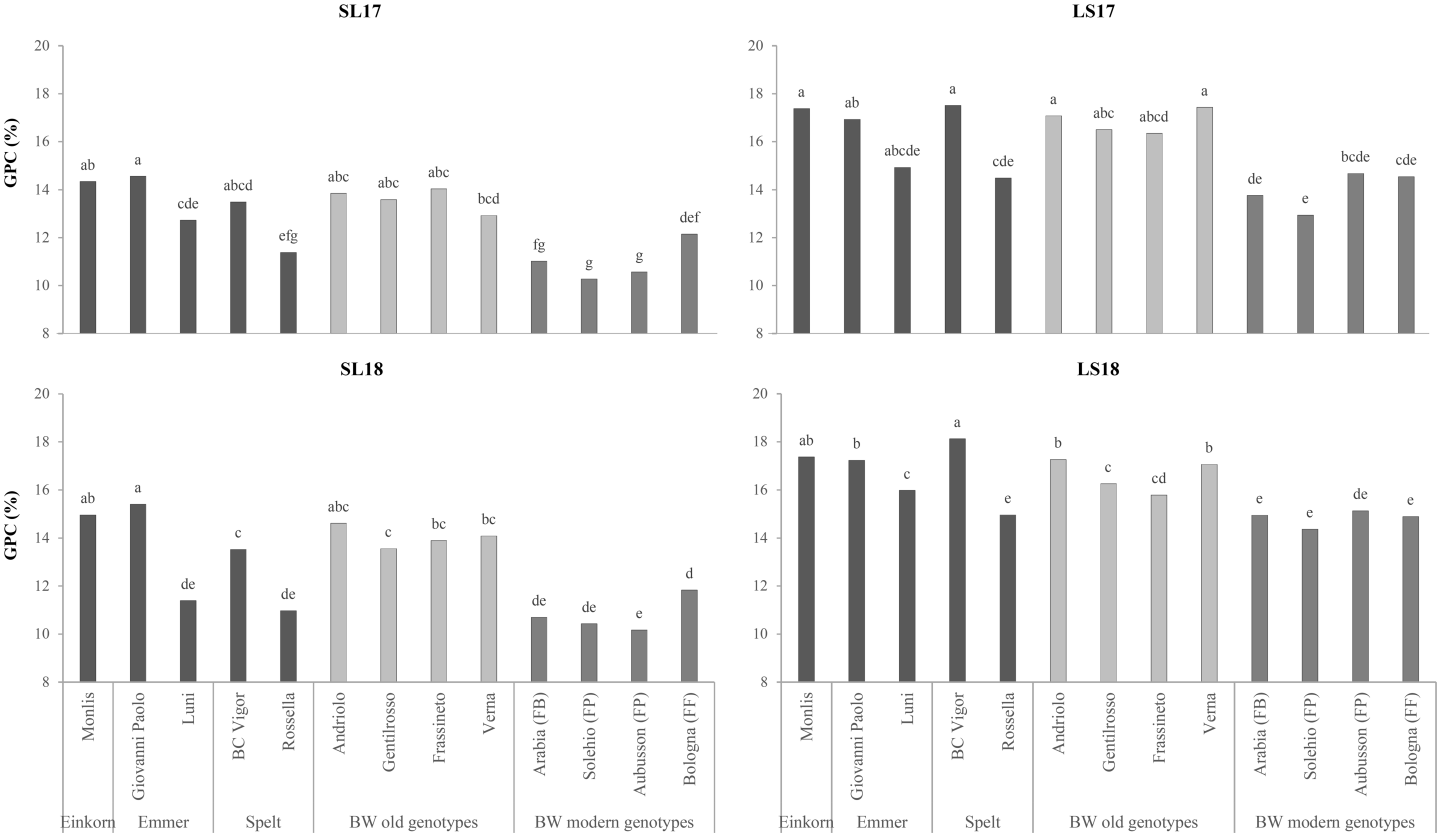
Comparison of the grain protein contents (GPCs) of the investigated genotypes across the growing environments. *SL17*, sandy-loam soil, harvested in 2017; *LS17*, loam-silty soil, harvested in 2017; *SL18*, sandy-loam soil, harvested in 2018; *LS18*, loam-silty soil, harvested in 2018; *BW*, bread wheat; *FB*, wheat for biscuits (frumento biscottiero); *FP*, ordinary bread-making wheat (frumento panificabile); *FF*, improver high-protein wheat (frumento di forza). Different letters above the columns indicate significant differences between the groups for *p* (*F*) < 0.05 according to the REGW-*F* test.

The einkorn presented the highest ear density, which led to the lowest TKW of all the genotypes (**Table 1**). Nevertheless, its TW was found to be the highest among the hulled wheat genotypes (71.7 *vs*. 63.3 kg hL^−1^). The TKW discriminated the hulled wheat and old BW wheat genotypes from the modern BW genotypes as the values were, on average, higher for spelt (+32%), emmer (+23%), and the old BW genotypes (+20%) than for the modern genotypes, while, as expected, spelt and emmer presented significantly lower TW values than the modern genotypes (−13% and −16%, respectively), among which the high-protein Bologna (FF) emerged (77.2 kg hL^−1^). The old BW genotypes were characterized by good TW values, similar to that of the ordinary bread-making Solehio (74.0 kg hL^−1^). The experiment factor had a significant effect on the ear density, and all of the studied genotypes showed a similar response between experiments, with a higher number of spikes per square meter where the fertility of the soil was greater (LS17 > LS18 > SL17 > SL18, with 89%, 73%, and 36% more than SL18). The TKW and TW traits showed differences among the experiments, with SL17 > SL18 > LS17 > LS18, although the genotypes behaved differently. The TW values of the old BW genotypes were always above the commercial threshold of 75 kg hL^−1^ in the SL17 and SL18 experiments, while the modern BW genotypes gave better results in 2017 (data not shown).The grains of all the genotypes belonging to the hulled wheat and the old BW genotypes were characterized by a much higher GPC than the grains of the modern genotypes (+17% as average values for both types), except for the emmer Luni, whose average content (13.7%) was similar to that of the high-protein Bologna (FF), and the spelt Rossella (12.6%), which was similar to that of the bread-making (FP) and biscuit-making (FB) wheat genotypes. The highest values were observed for the einkorn Monlis (16.1%), although the emmer Giovanni Paolo, the spelt BC Vigor, and the old BW Andriolo did not differ significantly. The ash content was significantly high in the einkorn (2.17%), the old BW genotypes (mean value of 2.03%), and the spelt BC Vigor (2.02%) grains, while low values were recorded for the modern BW genotypes (mean value of 1.88%). The GPC increased, albeit differently among the genotypes, along with the fertility of the soils, with +20% in LS17 and LS18 compared with SL18 and SL17 (**Figure 2**). The highest ash content was observed in the LS18 experiment, probably due to the lower recorded TW values and the consequent higher bran/endosperm ratio.

Estimates of the variances due to the G × E interaction (*σ*^2^_G×E_) were considerably smaller than the *σ*^2^_G_ for the agronomic and qualitative parameters across all genotypes. This resulted in high heritability estimates (**Table 1**), which ranged from 0.88 (ear density) to 0.99 (TKW).

**Figure 3:**
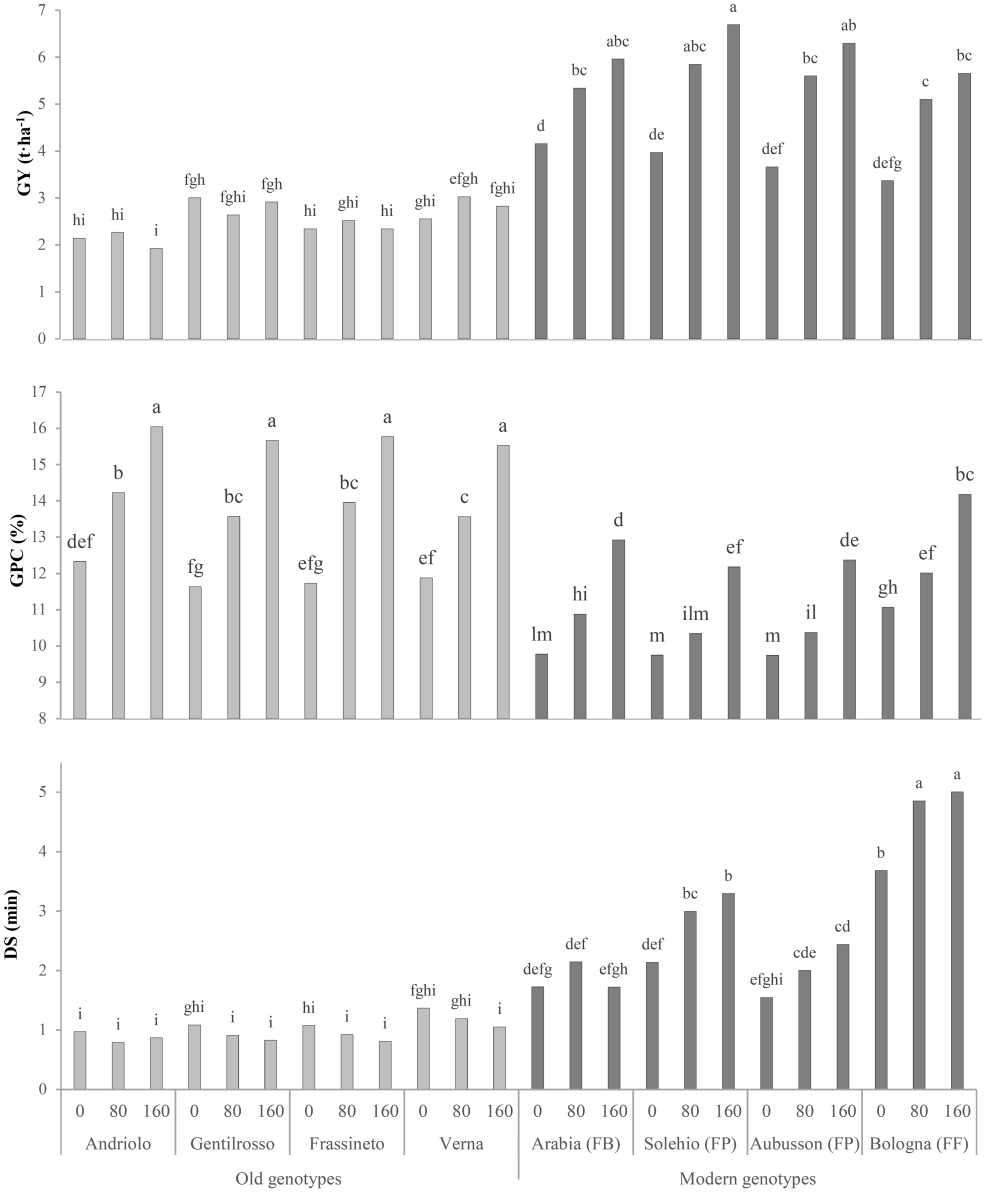
Comparison of the grain yields (GYs), grain protein contents (GPCs), and dough stability (DS) of the investigated bread wheat genotypes cultivated in the Cigliano experimental area and supplied at three different soil N rates (0, 80, and 160 kg N ha^−1^). *FB*, wheat for biscuits (frumento biscottiero); *FP*, ordinary bread-making wheat (frumento panificabile); *FF*, improver high-protein wheat (frumento di forza). Data are the average of 2 years. Different letters above the columns indicate significant differences between the groups for *p* (*F*) < 0.05 according to the REGW-*F* test.

The N effect was evident for all the tested genotypes (old and modern BW genotypes) and all the investigated traits (**Table 2**), with significantly higher values of GY (+47%), AUCGC (+27%), lodging (mainly pertaining to the old BW genotypes, with no significant difference in the N_80_ and N_160_ range), and GPC (+31%) from N_0_ to N_160_. Overall, better GY results were achieved in 2018 (+9%), which, however, was characterized by lower TKW, TW, and GPC values. The TKW increased for N_80_ and diminished for N_160_, but a differing N effect on this trait was found in relation to the genotype. The significant decrease observed for the TW along with the scale-up of the N rate was mainly observed for the old BW genotypes (3%–5% decline at N_160_ with respect to N_0_), as it remained the same or even rose slightly (0%–2%) in the modern ones (data not shown). The effect of the significant G × N interaction on GY and GPC is shown in **Figure 3**. The old BW genotypes had the same GY at N_0_, N_80_, and N_160_, and, in some cases, the increase in the N input rates determined yield losses, although not in a significant way. The old BW genotypes bred in the 20th century, i.e., Frassineto and Verna, showed an optimized productivity for N_80_, while the modern ones showed a maximum for N_160_. Indeed, except for the biscuit-making (FB) genotype, which showed a better yield, no significant differences were detected between the old and modern BW genotypes for N_0_. The modern BW genotypes, unlike the old ones, instead showed significant increases for N_80_ (+64%) and N_160_ (+84%). The GPC increased, albeit differently, among the genotypes along with the N supply. All of the genotypes exhibited a maximum level for N_160_, but the modern BW genotypes required the highest N application to reach similar values to those of the old genotypes with no N input, except for the modern high-protein (FF) genotype, which reached a similar level for N_80_ and further increased it for N_160_. The responses of the old genotypes to increasing N inputs were higher: the GPC increased, on average, by 16% and 8% for the old and modern BW genotypes, respectively, for N_80_, and by 32% and 28%, respectively, for N_160_.

**Table 2:** Effect of the genotype, the N fertilization rate, the harvest year, and their interaction on the agronomic traits and qualitative parameters of the investigated bread wheat genotypes cultivated in the Cigliano experimental area.

Factor	Bread wheat	Source of variation	GY (t ha^−1^)		AUCGC (NDVI-day)		Lodging (%)		TKW (g)		TW (kg hL^−1^)		GPC (%)	
Genotype (G)	Old genotypes	Andriolo	2.1	e	64.8	a	68.3	a	46.3	c	76.0	c	14.3	a
		Gentilrosso	2.8	cd	65.3	a	66.8	a	53.1	a	76.6	bc	13.7	b
		Frassineto	2.4	de	65.1	a	63.3	a	52.6	a	75.8	c	14.0	ab
		Verna	2.9	c	64.0	a	52.3	a	43.6	e	77.4	b	13.8	b
	Modern genotypes	Arabia (FB)	5.2	ab	64.6	a	0.2	b	45.0	d	74.3	d	11.2	d
		Solehio (FP)	5.6	a	64.6	a	0.0	b	49.5	b	76.1	c	10.8	e
		Aubusson (FP)	5.4	a	60.4	b	0.0	b	41.1	f	73.6	d	10.9	e
		Bologna (FF)	4.8	b	57.7	c	0.2	b	34.8	g	78.9	a	12.4	c
		*p* (F)	***		***		***		***		***		***	
N fertilization (N) (kg N ha^−1^)		0	3.2	c	53.7	c	3.9	b	45.5	b	76.7	a	10.9	c
		80	4.0	b	64.4	b	38.3	a	46.4	a	76.1	b	12.5	b
		160	4.4	a	68.4	a	42.2	a	45.3	b	75.7	c	14.3	a
		*p* (F)	***		***		***		***		***		***	
Harvest year (Y)		2017	3.9	a	62.0	b	40.1	a	47.3	a	76.7	a	12.8	a
		2018	3.9	b	64.3	a	24.9	a	44.6	b	75.6	b	12.5	b
		*p* (F)	***		***		ns		***		***		**	
G × N		*p* (F)	***		**		***		***		***		***	
G × Y		*p* (F)	ns		ns		ns		***		***		***	
N × Y		*p* (F)	**		***		ns		**		**		***	
G × N × Y		*p* (F)	ns		ns		ns		ns		***		ns	

Means followed by different letters are significantly different, according to the REGW-F test [(*) p (F) < 0.05, (**) p (F) < 0.01, (***) p (F) < 0.001, and ns, non-significant].

*GY*, grain yield; *AUCGC*, area under the canopy greenness curve; *TKW*, thousand kernel weight; *TW*, test weight; *GPC*, grain protein content; *FB*, wheat for biscuits (frumento biscottiero); *FP*, ordinary bread-making wheat (frumento panificabile); *FF*, improver high-protein wheat (frumento di forza).

#### 3.2.1 Rheological properties

The Mixolab^®^ parameters, which were obtained to investigate the mixing and viscous properties of the dough and to estimate the bread-making quality of the refined flour, were affected to a great extent by the genotype and the N fertilization (**Table 3**). The old BW genotypes did not differ significantly from the modern ones in terms of dough development time (DDT), and only the FF wheat (Bologna) emerged. The dough stability (DS) discriminated the old from the modern BW genotypes, with a net improvement of 189% on average observed in the latter ones, which reported differences according to the bread-making quality (a higher value in FF than in FP and FB). The high protein content of the old genotype flours could have led to doughs that were not sufficiently hydrated at 55% of WA, thereby resulting in higher mechanical stress, as indicated by the higher values of C1 (1.05 *vs*. 0.81 Nm for the old and modern BW genotypes), i.e., the maximum torque during mixing, which is an indication of insufficient dough plasticity due to a lack of hydration and, consequently, poor dough workability. The old genotypes were also characterized by lower values of C2 (data not shown), i.e., the minimum torque following kneading and temperature increase, and therefore by a significantly greater decrease in dough consistency due to the destabilization and unfolding of the proteins, which become hydrophobic, as reflected by the higher dough weakening (C1 − C2) parameter (0.63 *vs*. 0.35 Nm). Protein denaturation involves the release of large amounts of water, which can hinder the processability of the dough, and can negatively affect gas retention and the final bread volume. The increase in dough consistency caused by the hydration of the starch granules with the water released by the thermally degraded proteins was indicated by the starch gelatinization (C3 − C2) parameter, which was similar among the old BW genotypes and slightly higher than the modern ones (2.47 *vs*. 2.42 Nm), as the FB and FF modern genotypes performed poorly. The hot gel was found to be more stable for the modern BW genotypes than for the old ones, as indicated by the cooking stability (C3 − C4) parameter (0.57 *vs*. 0.48 Nm). C3 − C4 gives indications of the decrease in dough consistency under a constant heating, before cooling and starch retrogradation, which depends on the rate of enzymatic hydrolysis and affects the bread volume and its porosity. No significant differences were detected for the cooling setback (C5 − C4) parameter for any of the analyzed factors. All of the investigated traits progressively increased as a function of the N rate, from N_0_ to N_160_, except for the gelatinization and the cooking stability, which declined as the rate increased due to a higher protein content. All of the parameters showed higher values in 2017, except for dough weakening, for which no significant differences were observed. The significant G × N interaction for the DS trait exhibited an interesting response (**Figure 3**), as the old BW genotypes did not show different values for a rising N supply, while the majority of the modern genotypes performed better than the old ones for N_0_ and optimized this parameter for N_160_, except for the FB genotype, which showed a peak for N_80_.

**Table 3:** Effect of the genotype, the N fertilization rate, the harvest year, and their interaction on the rheological parameters of the refined flour of the investigated bread wheat genotypes cultivated in the Cigliano experimental area.

Factor	Bread wheat	Source of variation	DDT (min)		DS (min)		C1 (Nm)		C1 − C2 (Nm)		C3 − C2 (Nm)		C3 − C4 (Nm)		C5 − C4 (Nm)	
Genotype (G)	Old genotypes	Andriolo	1.1	b	0.9	d	1.1	ab	0.6	a	2.6	ab	0.4	de	0.9	a
		Gentilrosso	1.1	b	0.9	d	1.0	abc	0.6	a	2.4	abc	0.5	bcd	0.6	a
		Frassineto	1.1	b	0.9	d	1.0	ab	0.6	a	2.4	abc	0.6	b	0.6	a
		Verna	1.2	b	1.2	d	1.1	a	0.6	a	2.4	abc	0.4	e	0.8	a
	Modern genotypes	Arabia (FB)	1.2	b	1.9	c	0.9	c	0.5	b	2.3	c	0.5	bcde	0.8	a
		Solehio (FP)	1.2	b	2.9	b	0.7	d	0.3	b	2.6	a	0.8	a	0.8	a
		Aubusson (FP)	1.2	b	2.1	c	0.7	d	0.4	b	2.5	abc	0.6	bc	0.7	a
		Bologna (FF)	2.0	a	4.6	a	0.9	bc	0.3	b	2.3	bc	0.4	cde	0.9	a
		*p* (F)	***		***		***		***		*		***		ns	
N fertilization (N) (kg N ha^−1^)		0	1.0	c	1.7	b	0.8	c	0.4	b	2.5	a	0.6	a	0.7	a
		80	1.2	b	2.1	a	0.9	b	0.5	a	2.5	a	0.5	ab	0.7	a
		160	1.5	a	2.1	a	1.1	a	0.5	a	2.3	b	0.5	b	0.8	a
		*p* (F)	***		*		***		*		*		***		ns	
Harvest year (Y)		2017	1.5	a	2.1	a	1.0	a	0.5	a	2.5	a	0.8	a	0.8	a
		2018	1.1	b	1.9	b	0.9	b	0.5	a	2.4	b	0.4	b	0.7	a
		*p* (F)	**		*		***		ns		**		***		ns	
G × N		*p* (F)	**		***		ns		ns		ns		ns		ns	
G × Y		*p* (F)	***		ns		ns		ns		ns		***		ns	
N × Y		*p* (F)	ns		ns		ns		ns		ns		*		ns	
G × N × Y		*p* (F)	ns		*		ns		ns		ns		ns		ns	

Means followed by different letters are significantly different, according to the REGW-*F* test [(*) *p* (F) < 0.05, (**) *p* (F) < 0.01, (***) *p* (F) < 0.001, and ns, non-significant].

*DDT*, dough development time; *DS*, dough stability; *C1*, maximum torque (peak of 1.1 ± 0.05 Nm at 45 °C); *C1* − *C2*, dough weakening; *C3* − *C2*, gelatinization; *C3* − *C4*, cooking stability; *C5* − *C4*, cooling setback; *FB*, wheat for biscuits (frumento biscottiero); *FP*, ordinary bread-making wheat (frumento panificabile); *FF*, improver high-protein wheat (frumento di forza).

### 3.3 Gluten immune toxicity

The quality of the investigated genotypes was also evaluated in terms of gluten immune toxicity by targeting potential toxic celiac-related epitopes with the R5 monoclonal antibody (**Table 4**). The R5 reactivity varied significantly among the genotypes, with no clear discrimination between the emmer, spelt, and modern wheat genotypes. Overall, the highest concentrations were observed in the old BW genotypes, with Andriolo presenting the maximum value (266 mg kg^−1^). The modern BW genotypes showed more heterogeneous values, and they did not differ significantly from the old genotypes, with the sole exception of the FP Solehio, which had the lowest absolute concentrations of R5 gliadins (133 mg kg^−1^). Despite their high protein content, the einkorn Monlis and the emmer Giovanni Paolo had a low R5 reactivity, while the emmer Luni showed a similar concentration to those of some BW genotypes.

**Table 4:** Effect of the genotype, the experiment, and their interaction on the R5 gliadin content of the refined flour and on the DON content, the bioactive compounds, and the antioxidant capacity of the wholemeal flour of the investigated genotypes cultivated in the experimental trials and supplied with 80 kg N ha^−1^.

Factor	Species		Source of variation	R5 gliadins (mg kg^−1^)		DON (µg kg^−1^)		SPAs (mg kg^−1^)		CWBPAs (mg kg^−1^)		ARs (mg kg^−1^)		AC_DPPH_ (mmol TE kg^−1^)		AC_FRAP_ (mmol TE kg^−1^)	
Genotype (G)	Einkorn		Monlis	150	de	7,798	bc	110	bc	668	ab	770.3	de	3.9	abc	8.8	bcde
	Emmer		Giovanni Paolo	178	cde	9,891	a	79	fg	551	cd	809.0	cde	3.6	ef	8.1	ef
			Luni	251	ab	5,851	cd	112	b	536	d	878.1	bcd	3.7	bcdef	7.6	fg
	Spelt		BC Vigor	244	abc	5,377	d	71	gh	666	ab	939.0	bc	3.8	bcde	8.1	def
			Rossella	179	bcde	9,485	ab	72	gh	604	bcd	672.5	e	3.6	def	9.2	abc
	BW	Old genotypes	Andriolo	266	a	2,326	ef	94	de	635	abc	772.4	de	3.8	abcd	9.6	ab
			Gentilrosso	227	abc	1,504	ef	98	de	654	ab	719.0	de	3.9	ab	9.7	a
			Frassineto	234	abc	1,179	f	99	cd	639	ab	774.7	de	3.9	abc	9.0	abc
			Verna	260	ab	1,323	f	130	a	609	bc	758.0	de	3.7	bcdef	9.0	abcd
		Modern genotypes	Arabia (FB)	239	abc	6,663	cd	66	h	610	bc	946.0	b	3.6	f	8.5	cde
			Solehio (FP)	133	e	2,415	ef	87	ef	704	a	758.1	de	3.8	abcde	9.5	ab
			Aubusson (FP)	217	abcd	3,472	e	63	h	647	ab	1,307.7	a	3.8	bcdef	9.6	ab
			Bologna (FF)	263	ab	1,039	f	68	gh	682	ab	1,408.9	a	4.0	a	7.3	g
			*p* (F)	***		***		***		***		***		***		***	
Experiment (E)			SL17	230	b	244	c	52	c	502	c	797.3	c	3.6	c	8.0	c
			LS17	275	a	769	c	57	c	514	c	859.4	b	3.7	b	8.3	c
			SL18	170	c	6,957	b	117	b	722	b	950.6	a	4.0	a	9.1	b
			LS18	201	b	9,045	a	129	a	793	a	970.3	a	4.0	a	9.6	a
			*p* (F)	***		***		***		***		***		***		***	
G × E			*p* (F)	***		***		***		***		***		***		***	
*H* ^2^				0.65		<0.01		0.83		<0.01		0.94		<0.01		0.42	

Means followed by different letters are significantly different, according to the REGW-F test [(*) *p* (F) < 0.05, (**) *p* (F) < 0.01, (***) *p* (F) < 0.001, and ns, non-significant]. Broad-sense heritability (*H*^2^) was estimated across genotypes and experiments.

*DON*, deoxynivalenol; *SPAs*, soluble phenolic acids; *CWBPAs*, cell wall-bound phenolic acids; *ARs*, 5-*n*-alkylresorcinols; *AC*, antioxidant capacity (DPPH and FRAP assays); *BW*, bread wheat; *FB*, wheat for biscuits (frumento biscottiero); *FP*, ordinary bread-making wheat (frumento panificabile); *FF*, improver high-protein wheat (frumento di forza); *SL17*, sandy-loam soil, harvested in 2017; *LS17*, loam-silty soil, harvested in 2017; *SL18*, sandy-loam soil, harvested in 2018; *LS18*, loam-silty soil, harvested in 2018.

### 3.4 Mycotoxin contamination

**Figure 4:**
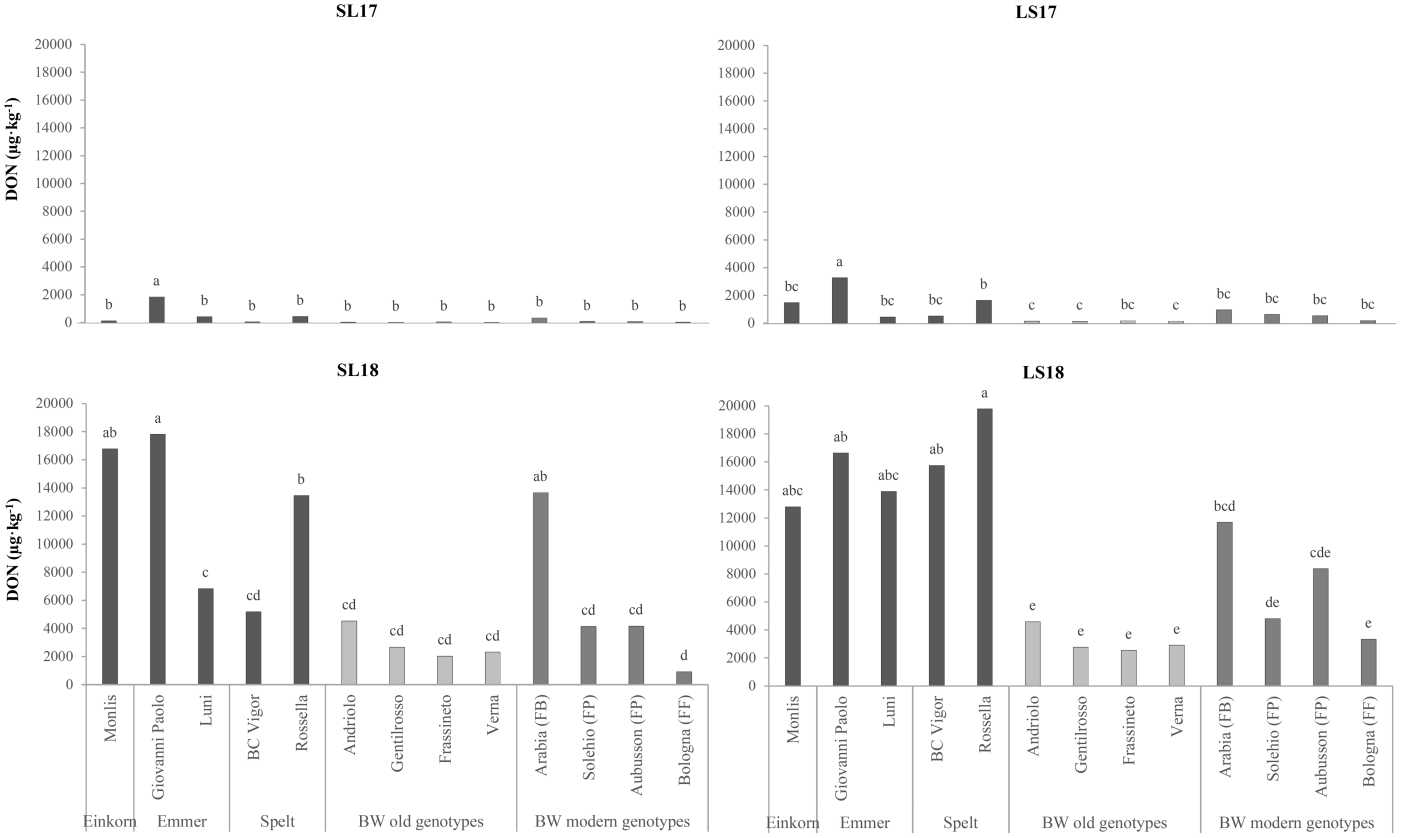
Comparison of the deoxynivalenol (DON) contents of the investigated genotypes across the growing environments. *SL17*, sandy-loam soil, harvested in 2017; *LS17*, loam-silty soil, harvested in 2017; *SL18*, sandy-loam soil, harvested in 2018; *LS18*, loam-silty soil, harvested in 2018; *BW*, bread wheat; *FB*, wheat for biscuits (frumento biscottiero); *FP*, ordinary bread-making wheat (frumento panificabile); *FF*, improver high-protein wheat (frumento di forza). The results are expressed on a dry weight (DW) basis. Different letters above the columns indicate significant differences between the groups for *p* (*F*) < 0.05 according to the REGW-*F* test.

Mycotoxin contamination was evaluated in terms of the DON content, which was influenced significantly by both the genotype and the experiment factors (**Table 4**), with concentrations that were 37 and 28 times higher in LS18 and SL18 than in SL17. Although the modern BW genotypes were more contaminated overall, the DON content of the FP and FF genotypes did not differ significantly from that of the old BW genotypes, while only the FB Arabia showed higher susceptibility (**Figure 4**). The hulled wheat genotypes resulted as the most susceptible to DON accumulation in all the experiments, and particularly in the growing environments characterized by favorable weather conditions for FHB infection. The emmer Giovanni Paolo showed the greatest DON content in all experiments and always exceeded the maximum level of DON set for unprocessed cereals (1,000 µg kg^−1^) (European Commission, 2023). The limit was exceeded in all the genotypes in the LS18 experiment, and the high-protein (FF) Bologna was the only genotype that presented a content under the maximum level in SL18.

### 3.5 Phytochemical compounds and antioxidant capacity

**Figure 5:**
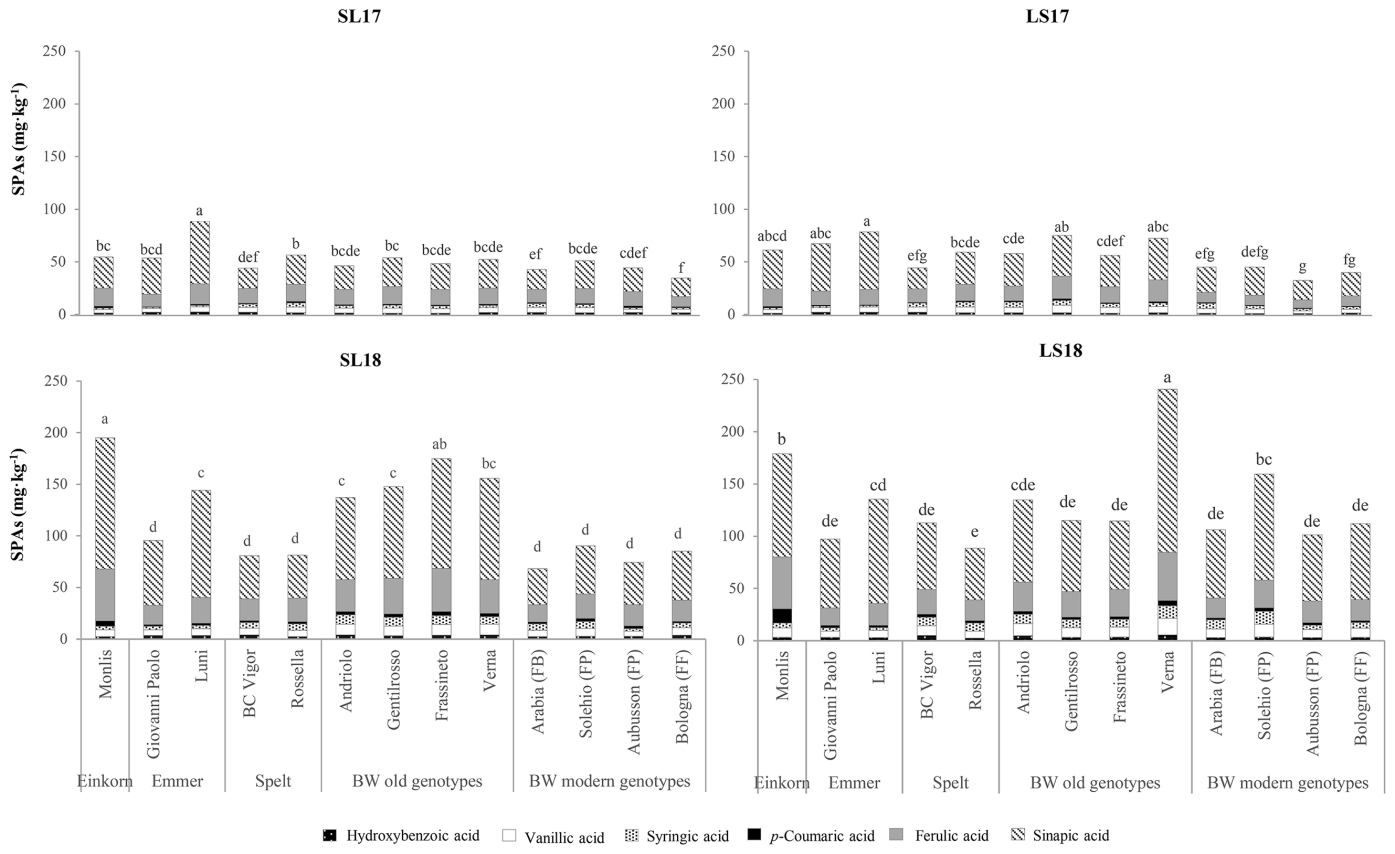
Comparison of the soluble phenolic acid (SPA) profiles of the investigated genotypes across the growing environments. *SL17*, sandy-loam soil, harvested in 2017; *LS17*, loam-silty soil, harvested in 2017; *SL18*, sandy-loam soil, harvested in 2018; *LS18*, loam-silty soil, harvested in 2018; *BW*, bread wheat; *FB*, wheat for biscuits (frumento biscottiero); *FP*, ordinary bread-making wheat (frumento panificabile); *FF*, improver high-protein wheat (frumento di forza). The results are expressed on a dry weight (DW) basis. Different letters above the columns indicate significant differences between the groups for *p* (*F*) < 0.05 according to the REGW-*F* test.

**Table 4** shows the health-related compounds of the wholemeal flour of the analyzed genotypes across the experiments. All parameters were affected significantly by the genotype and experiment factors, as well as by their interactions. Majority of the differences among the genotypes emerged from the SPA fraction, although its contribution to the total content was the lowest, ranging from 10% in the spelt and modern BW genotypes to 15% in the einkorn, emmer, and the old BW genotypes. The highest share (18%) and content (130 mg kg^−1^) of SPAs were observed in the old genotype Verna, and this was followed by the einkorn Monlis and the emmer Luni (average value of 111 mg kg^−1^). The modern BW genotypes presented the lowest SPA content (average value of 71 mg kg^−1^) and were not significantly different from those of the spelt, except for Solehio, whose content was lower but statistically equal to those of the old genotypes Andriolo and Gentilrosso (average value of 96 mg kg^−1^). The CWBPA concentrations of the investigated genotypes showed less marked differences. The modern BW genotypes showed the highest values, but neither the old genotypes nor the spelt and einkorn wheat genotypes were significantly different from them. As expected, sinapic acid was the most abundant phenolic acid in the soluble form (average value of 52 mg kg^−1^, ranging from 51% to 71% of the total SPA content) (**Figure 5**), while ferulic acid made up majority of the bound fraction (543 mg kg^−1^, ranging from 79% to 88% of the total CWBPA content; data not shown). The second predominant compound in the soluble form was ferulic acid (20 mg kg^−1^, 18%–26%), followed by vanillic (6.4 mg kg^−1^, 5%–9%), syringic (4.6 mg kg^−1^, 2%–9%), hydroxybenzoic (2.7 mg kg^−1^, 2%–5%), and *p*-coumaric acid (2.2 mg kg^−1^, 2%–4%) (**Figure 5**), while the CWBPA composition was less heterogeneous (data not shown), with sinapic acid accounting for 4%–9% of the total CWBPA content (average value of 43 mg kg^−1^), followed by *p*-coumaric acid (29 mg kg^−1^, 3%–12%). The bound caffeic, vanillic, syringic, and hydroxybenzoic acids accounted for less than 1% each. Qualitative differences in the SPA and CWBPA composition emerged across the genotypes. Einkorn showed the highest concentration of *p*-coumaric acid (both soluble and bound) and sinapic acid (only bound). The SPA content and the composition of the emmer genotypes were different from each other, with Luni showing the highest total amount in all the environments and higher concentrations of ferulic, sinapic, and vanillic acids. The old BW genotypes had above average soluble ferulic, synapic, syringic, and vanillic acid contents, while the soluble and bound phenolic acid compositions of the modern BW genotypes were more heterogeneous. The SPAs and CWBPAs showed a similar response to the varying growing conditions, with larger concentrations recorded in the LS18 (+150% and +58%) and SL18 (+126% and +44%) experiments than in the LS17 and SL17 ones.

The average AR content (**Table 4**) varied significantly among the analyzed genotypes, with higher levels being observed in the modern BW than in the old genotypes. The modern genotypes Bologna and Aubusson presented the highest concentrations (average of 1,358.3 mg kg^−1^), while the spelt Rossella showed the lowest (672.5 mg kg^−1^). The old BW genotypes displayed similar values to that of the aforementioned spelt (average of 756.0 mg kg^−1^). The experiments conducted in 2018 resulted in a significantly higher AR accumulation than LS17 (−11%) and SL17 (−18%).

The DPPH and FRAP assays were used to evaluate the *in vitro* AC of the flour samples (**Table 4**) by measuring, respectively, the scavenging of the DPPH radicals and the reduction of Fe^3+^ to Fe^2+^, leading to the concentration of electron-donating antioxidants (Serpen et al., 2012). The old genotype Gentilrosso exhibited superior AC in both assays, while the other old BW genotypes and the modern bread-making (FP) genotype were characterized by lower but statistically similar values. The significantly high AC_DPPH_ registered for the einkorn (Monlis) and the FF modern wheat (Bologna) was not consistent with the poor AC_FRAP_ values. As far as environment is concerned, the AC generally showed a similar response to that found for the investigated phenolic compounds (LS18 > SL18 > LS17 > SL17). The AC determined by both assays was moderately correlated with both the SPA (Pearson’s correlation coefficient: *r*^2^ = 0.46, *p* < 0.01, both AC_DPPH_ and AC_FRAP_) and the CWBPA contents (0.58, *p* < 0.01 for AC_DPPH_; 0.47, *p* < 0.01 for AC_FRAP_). A modest but significant correlation of the AR content was only observed for AC_DPPH_ (0.29; *p* < 0.01). Indeed, the amphiphilic AR molecules were more likely to be detected in the semi-organic methanol/water (50:50, *v/v*) mixture, in which the DPPH radicals were dissolved, than in the FRAP reaction medium, which was an aqueous acetic buffer used to maintain iron solubility.

Relatively large estimates of *σ*^2^_G_ compared with *σ*^2^_G×E_ across all genotypes were determined for the SPAs and ARs, resulting in high heritability. In contrast, the other investigated traits (R5 gliadins, DON, CWBPAs, and AC) were characterized by low heritability estimates (**Table 4**).

## 4 Discussion

By comparing 13 *Triticum* spp. genotypes, including einkorn, emmer, spelt, and old and modern BW genotypes, under standard agronomic practices, the field experiments highlighted the role of pedoclimatic conditions on a large set of quantitative and qualitative traits. The abundant rainfall observed in 2017–2018 adversely affected the yield and quality traits. In particular, the seasonal heavy rains were largely responsible for the resulting low yield of LS18, despite the good soil fertility, as they facilitated N-soil leaching, plant lodging, and fungal infection. The higher FHB infection, in particular of the modern BW genotypes, and the consequent grain shriveling in 2018 affected the milling and the safety of the grains in terms of the TW values and the DON content.

The yield differences between the hulled wheat and modern BW genotypes were consistent with previous experiments conducted under conventional management conditions, where the mean GYs of spelt, emmer, and einkorn were 40%, 55%, and 62% lower, respectively, than those of BW (Longin et al., 2016; Rachoń et al., 2020). The superior yields of the modern BW genotypes under favorable conditions (**Figure 1**) and their responsiveness to the N inputs (**Figure 3**) confirm their greater yield potential and their optimization for high-input systems. In contrast, the higher yield stability of the old BW genotypes under diverse conditions did not translate into superior performance in low-yielding environments, as previously reported by other authors (Acreche et al., 2008; Ferrante et al., 2017; Guarda et al., 2004). The better adaptation of spelt to the low-nutrient conditions suggests its potential use in marginal environments. The lodging susceptibility of old BW and some hulled wheat genotypes highlights the importance of plant height in determining agronomic performance, especially when grown under conditions of high soil fertility (LS17 experiment). The lower plant height and the higher HI of the modern BW genotypes reflect the ongoing breeding improvements, while the extensive variability in HI among modern hulled wheat genotypes indicates the need for the further optimization of these genotypes.

The potential of producing high protein and ash contents under the same growing conditions is in line with previous reports in low-input production situations for einkorn, emmer, and spelt (Longin et al., 2016; Golea et al., 2023) and for old BW genotypes (Migliorini et al., 2016; Simsek et al., 2019), affirming their value for nutritional and specialty markets. The lower protein and ash contents of the modern BW genotypes have been attributed to dilution effects following a selection for starch accumulation in order to increase the grain size and productivity (Fan et al., 2008; Shewry and Hey, 2015), although also the no effect on the GPC of the old and modern BW genotypes was observed (Simsek et al., 2019). GPC is one of the qualitative parameters used to commercially classify BW flour into quality categories for industrial processing (Foca et al., 2007), and the ash content is an indicator of the mineral content of the grain, which is essential for both human nutrition and for the leavening properties of the dough. However, bread-making quality hinges not only on the protein quantity but also on its composition, which can be explored through empirical rheological analyses.

Both the old and modern BW genotypes were grown under N-input regimes of 0, 80, and 160 kg N ha^−1^ in the same location (Cigliano, on sandy-loam soil) over the two growing seasons. This approach was aimed at further elucidating the agronomic and bread-making qualities of these genotypes within the context of a high-input production system. The differential response to the N input between the old and modern BW genotypes underlines the higher efficiency of the modern ones in utilizing higher N rates for productivity and yield components. The similar or decreasing yields of the old BW genotypes at higher N inputs suggest limitations in their nutrient assimilation capacity, aligning with previously reported results (Guarda et al., 2004). The higher GPC of the old BW genotypes at minimal N inputs reinforces their potential in supply chains that prioritize nutritional grain quality over yield, although it opens up investigations into rheological quality for food applications. Complex rheological characterization using Mixolab^®^ provides information on both the mixing, mainly related to the gluten quality, and the carbohydrate-dependent viscous properties and can thus provide more detailed information on the technological usability of old BW genotypes (Bánfalvi et al., 2020). The rheological analysis confirmed that modern BW genotypes are better suited for bread-making, with superior DS and workability compared with the old genotypes. The weaker dough networks and the suboptimal pasting behavior of the old genotypes, even after the application of N fertilizer, may limit their stand-alone use in bread-making, but offer opportunities for blending with stronger flours or employing tailored processing techniques. The data reported in the literature point out that the decreasing trend in the protein concentration of grains over time was nevertheless accompanied by an enhanced quality of the composition (Ribeiro et al., 2016) and rheological traits (Guarda et al., 2004; Ormoli et al., 2015; Mefleh et al., 2022), such as in the *W* and *P*/*L* indices of the alveograph and of the sodium dodecyl sulfate (SDS) sedimentation volume. The response of the Mixolab^®^ parameters to the increasing N supply in the modern BW genotypes supports the efforts made in breeding for improving both yield and bread-making quality, which are also evident for no N supply. In addition, a greater variability was found among the modern BW genotypes, which could thus be selected for specific purposes and diverse food applications.

The findings of this study indicate that the immunogenic potential of gluten proteins, as measured by means of R5 gliadin quantification, is highly genotype-dependent and is not significantly higher in modern wheat compared with the old genotypes. This challenges the hypothesis that modern breeding practices, aimed at enhancing the gluten properties, have led to increased immunotoxicity. The relatively low response toward the R5 monoclonal antibody observed in some einkorn and emmer genotypes suggests that these hulled wheat genotypes may offer lower risks of celiac-related immune responses, consistent with the absence of the D genome in these species (Sharma et al., 2020). However, the variability among the emmer genotypes highlights the complexity of this relationship and requires further investigation. Interestingly, the modern BW genotypes such as Solehio, despite the improved protein quality, had lower immunogenic gliadin proteins, supporting the notion that breeding has predominantly targeted glutenins, which are mainly responsible for dough strength and are less immunogenic than gliadins (Sharma et al., 2020). These results align with studies showing that hulled wheat and old BW genotypes, with the exception of *T. monococcum*, can produce a larger number of peptides containing immunogenic and toxic sequences after digestion (Ficco et al., 2019; Prandi et al., 2017).

The data on DON contamination emphasize the influence of both genotype and environmental factors. The lower susceptibility of the old BW genotypes to DON accumulation may be attributed to their taller plant structure, which physically impedes *Fusarium* conidia infection (Chrpová et al., 2013), and to possible biochemical mechanisms of resistance (Dong et al., 2023). Contrary to prior studies suggesting higher DON levels in modern BW genotypes under artificial inoculation (Wiwart et al., 2016), our findings revealed substantial variability in the occurrence of DON among genotypes under natural infection conditions. Indeed, the modern genotype Bologna (FF) had the consistently lowest level of mycotoxin contamination within all the compared production situations.

The higher SPA levels in the old BW genotypes, especially in Verna, corroborate earlier reports that these genotypes grown under low-input management may contain higher and more diverse polyphenols compared with modern ones (Di Silvestro et al., 2012; Dinelli et al., 2011; Montevecchi et al., 2019). Nevertheless, high contents of total phenolics (Dinelli et al., 2011) and ferulic acid (Qayyum et al., 2024) have also been reported for modern BW genotypes in low- and high-input production situations, respectively. The modern BW genotypes in this study demonstrated significant variability, with some (Solehio) showing phenolic levels comparable to those of the old BW genotypes and others, such as Bologna, excelling in antioxidant capacity (AC_DPPH_) and levels of AR phenolic lipids. Other authors have also investigated the content and the composition of phenolic compounds in both hulled and non-hulled wheat species, although the findings are often inconsistent. Li et al. (2008) reported the highest total phenolic content in emmer, followed by einkorn and spelt, whereas Brandolini et al. (2013) observed the opposite trend. Compared with BW, emmer has often exhibited higher total phenolic levels (Györéné Kis et al., 2025; Lacko-Bartošová et al., 2023; Li et al., 2008; Zrcková et al., 2019). When analyzing the phenolic acids in the soluble and bound forms, Benincasa et al (2015) noted significantly higher levels of CWBPAs in einkorn and emmer compared with spelt and BW, while the emmer genotypes of the present study exhibited lower values. Barański et al. (2020) reported that almost all SPAs were significantly higher in einkorn than in emmer and spelt, similarly to what we observed. However, marked differences were observed for the two emmer genotypes in the present study. The variation in the phenolic compounds such as phenolic acids and ARs is often attributable to their localization in the testa and pericarp of wheat kernels (Pedrazzani et al., 2021), which makes the total content susceptible to differences in the kernel size among genotypes and negatively correlated with the TKW. Based on the AC assays performed and the phytochemical compounds quantified, neither the old nor the modern BW genotypes appear to be uniformly superior in health-related traits. These results are consistent with previous studies that reported no significant decline in the AC_ABTS_ of durum wheat due to breeding efforts (Laus et al., 2015), as well as no major differences at the nutritional and phytochemical levels between the hulled species (einkorn, emmer, and spelt) and the modern BW genotypes (Shewry and Hey, 2015; Spisni et al., 2019), with only einkorn, emmer, and khorasan showing high concentrations of the carotenoid lutein (Ziegler et al., 2016).

Environmental factors also played a prominent role. Experiments in the 2018 harvest (LS18 and SL18) were characterized by above-average rainfall levels and a pronounced grain shriveling. These experiments yielded higher AC and phenolic concentrations, consistent with the changes in the kernel parameters and the higher presence of bran, where notably higher concentrations of antioxidant compounds were found. The observed inter-experiment variations were primarily influenced by the modern BW genotypes, which appeared to generally be less stable across experiments due to their improved responsiveness to the varying production conditions, with consequent changes in grain traits such as kernel size and grain composition. Environmental factors such as temperature and precipitation during grain maturation can affect the phenolic concentration levels across growing seasons, e.g., higher rainfall was associated with increased phenolic acid content (Györéné Kis et al., 2025; Rachoń et al., 2020), while extremely dry and hot weather during the maturity stages was associated with lower levels of soluble and bound phenolic acids (Lacko-Bartošová et al., 2023). However, due to the absence of comprehensive literature reviews or meta-analyses and the high degree of methodological heterogeneity in existing studies (Tian et al., 2022), definitive conclusions remain tentative.

**Figure 6:**
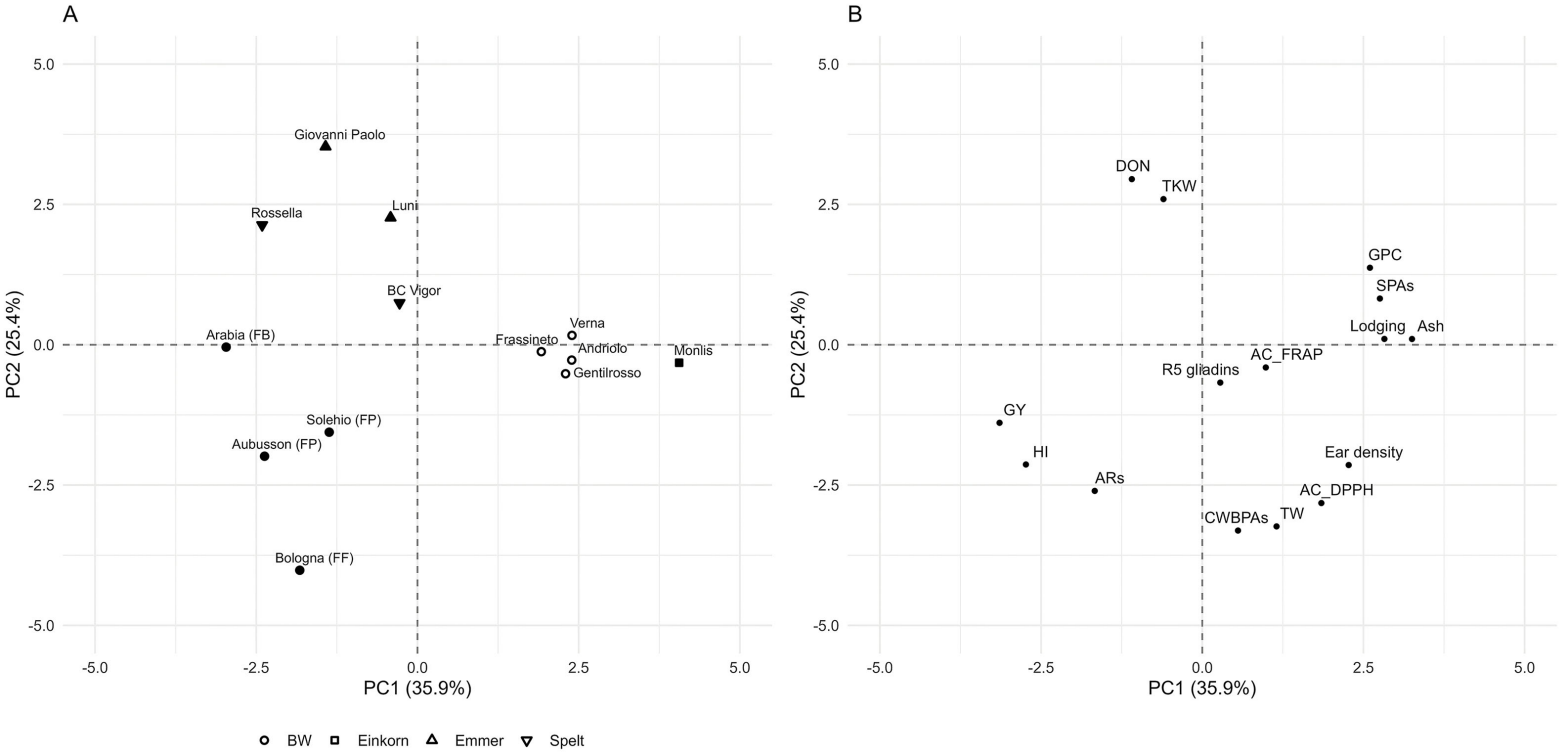
Multivariate analysis of the agronomic and qualitative parameters of the 13 genotypes grown in four environments. **(A)** Score plot of the principal component analysis (PCA). **(B)** Loading plot for the separation in **(A)**. The sample plots are grouped on the basis of the average mean of three replications and four experiments. *Different shapes* correspond to the crop type shown in the legend. *Open symbols* represent the old genotypes, while *filled symbols* indicate the modern genotypes (released after 2000). The loadings of the variables of PC1 and PC2 are shown in **Supplementary Figure S1**. *BW*, bread wheat; *FB*, wheat for biscuits (frumento biscottiero); *FP*, ordinary bread-making wheat (frumento panificabile); *FF*, improver high-protein wheat (frumento di forza); *AC*, antioxidant capacity (DPPH and FRAP assays); *ARs*, 5-*n*-alkylresorcinols; *CWBPAs*, cell wall-bound phenolic acids; *DON*, deoxynivalenol; *GPC*, grain protein content; *GY*, grain yield; *HI*, harvest index; *SPAs*, soluble phenolic acids; *TKW*, thousand kernel weight; *TW*, test weight.

Further insight into the differences in the agronomic and qualitative traits among the 13 genotypes was provided by multivariate analysis. The PCA (**Figure 6**) allowed for partial separation among crop types and between the modern and old BW genotypes. The modern genotypes of emmer, spelt, and BW were all positioned on the left side of the graph, mainly differentiated along PC2 (**Figure 6A**). The loadings plot (**Figure 6B**) indicated that the old BW genotypes and einkorn were particularly associated with higher lodging susceptibility, as well as greater protein, ash, and SPA contents. Overall, the multivariate approach supported the observed variation in both the agronomic performance and the compositional profiles, particularly highlighting the distinctiveness of old BW genotypes and the different profiles of modern emmer, spelt, and BW genotypes.

## 5 Conclusions

The comparative study revealed the agronomic challenges associated with growing both hulled species and old BW genotypes in non-marginal cropping systems. The selected old BW genotypes demonstrated diminished adaptability to diverse environmental conditions and limited responsiveness to improved production conditions. Their grains had a significantly higher protein content than those of the modern genotypes, but their dough presented greater processing difficulties, even with increased rates of nitrogen fertilization. The old BW genotypes offered no advantage in terms of toxic celiac disease epitopes, but exhibited a reduced susceptibility to mycotoxin accumulation. However, genotypes less affected by the contamination were also found within the hulled and the modern bread wheat groups. The old BW genotypes had slightly higher phenolic acids but lower phenolic lipids than the modern BW genotypes, while the hulled species showed variable profiles. Overall, differences in health-related traits were quite modest and were dependent on the growing conditions, so that, overall, they might not be considered relevant in the context of human diets. In addition, a higher level of biodiversity is generally pointed out as a strength of landraces and old BW genotypes. On the basis of the limited, albeit representative, number of genotypes that we analyzed in different production situations, a clearly greater variability of many of the studied traits was observed for the modern BW genotypes than for the old ones. Furthermore, the recently released hulled wheat genotypes, despite still suffering from agronomic deficiencies, have exhibited a wide range for all the agronomic and qualitative traits. This emphasizes the great importance of targeted breeding and varietal selection, to allow the progressive identification of new genotypes, as they are capable of ensuring high yields and technological performances, even with a low N supply. Moreover, these can be further improved in terms of safety and phytochemical quality, in accordance with the requirements of the food supply chain.

## Data Availability

The raw data supporting the conclusions of this article will be made available by the authors, without undue reservation.
