# Supplementary material for: Growing hulled wheat and old bread wheat genotypes in non-marginal environments: agronomic, qualitative, and safety insights
Source: Front Plant Sci. 2025 Nov 6;16:1638426. doi: 10.3389/fpls.2025.1638426 (PMC12631291; doi:10.3389/fpls.2025.1638426)
Supplement: Supplementary file 1 [file DataSheet1.docx]

**Table S1.** The hulled (einkorn, emmer, and spelt) and old and modern bread wheat (BW) genotypes compared in this study.

| **Crop** | **Taxonomic classification** | **Ploidy level^a^** | **Type^b^** | **Accession^c^** | **Plant height (cm)^d^** | **Origin** | **Year of release** |
| --- | --- | --- | --- | --- | --- | --- | --- |
| Einkorn | *T. monococcum* spp. *monococcum* | Diploid  (AA) | Hulled genotypes | Monlis | 122 - 172 | CREA-IT-RM, Rome (RM), Italy | 2006 |
| Emmer | *T. turgidum* spp. *dicoccum* | Tetraploid (AABB) |  | Giovanni Paolo | 78 - 141 | CREA-CI-FG, Foggia (FG), Italy | 2008 |
|  |  |  |  | Luni | 103 - 175 | S.I.S. S.p.A., San Lazzaro di Savena (BO), Italy | 2002 |
| Spelt | *T. aestivum* spp. *spelta* | Hexaploid (AABBDD) |  | BC Vigor | 128 - 184 | Bc Institut, d.d., Zagreb, Republika Hrvatska | 2012 |
|  |  |  |  | Rossella | 103 - 151 | CREA-CI-FG, Foggia (FG), Italy | 2016 |
| BW | *T. aestivum* spp*. aestivum* | Hexaploid (AABBDD) | Old genotypes | Andriolo | 119 - 173 | Tuscany, Italy | From the 19^th^ century |
|  |  |  |  | Gentilrosso | 128 - 174 | Tuscany, Italy | From the 19^th^ century |
|  |  |  |  | Frassineto | 113 - 164 | Tuscany, Italy | 1922 |
|  |  |  |  | Verna | 124 - 182 | Tuscany, Italy | 1953 |
|  |  |  | Modern genotypes | Arabia (FB) | 78 - 105 | Apsovsementi S.p.A., Voghera (PV), Italy | 2009 |
|  |  |  |  | Solehio (FP) | 75 - 106 | Agroalimentare Sud S.p.A., Melfi (PZ), Italy | 2008 |
|  |  |  |  | Aubusson (FP) | 49 - 97 | Limagrain Italia S.p.A., Fidenza (PR), Italy | 2003 |
|  |  |  |  | Bologna (FF) | 70 - 91 | S.I.S. S.p.A., San Lazzaro di Savena (BO), Italy | 2002 |

^a^Number of the chromosome sets. ^b^Andriolo, Gentilrosso, Frassineto, and Verna are historically cultivated bread wheat genotypes (from before 1985); Andriolo and Gentilrosso are landraces, that is, genetically heterogenous populations that have been cultivated from the 19^th^ century, while Frassineto and Verna are old genotypes that were developed from varietal crosses; the einkorn, emmer, spelt, and the other bread wheats are modern genotypes that were released after 2000. ^c^FB: wheat for biscuits (frumento biscottiero); FP: ordinary bread-making wheat (frumento panificabile); FF: improver high protein wheat (frumento di forza). ^d^The minimum and maximum plant heights measured in the experimental trials.

**Table S2.** The physical and chemical characteristics of the soils of the field experiments conducted in the 2016-18 period in north-west Italy.

|  |  | **2016-17** | | **2017-18** | |
| --- | --- | --- | --- | --- | --- |
|  |  | **Cigliano** | **Carmagnola** | **Cigliano** | **Carmagnola** |
| **Parameter** | **Measuring unit** | **SL17** | **LS17** | **SL18** | **LS18** |
| Geographic coordinates | | 45° 18’ N, 8° 01’ E | 44° 50’ N, 7° 40’ E | 45° 18’ N, 8° 01’ E | 44° 50’ N, 7° 40’ E |
| Altitude | m | 236 | 245 | 236 | 245 |
| Soil (USDA classification) | | Typic Hapludalfs | Typic Udifluvents | Typic Hapludalfs | Typic Udifluvents |
| Sand (2 -0.05 mm) | % | 41.7 | 27.7 | 44.8 | 28.6 |
| Silt (0.05 - 0.002 mm) | % | 47.5 | 65.4 | 45.9 | 64.8 |
| Clay (< 0.002 mm) | % | 10.8 | 6.9 | 9.3 | 6.5 |
| pH |  | 5.9 | 7.9 | 5.7 | 8.1 |
| Organic matter | % | 1.51 | 1.62 | 1.69 | 1.44 |
| C/N |  | 9.4 | 8.3 | 12.0 | 8.0 |
| Cation Exchange Capacity (C.E.C.) | Cmol(+)·kg^-1^ | 10.2 | 10.7 | 11.2 | 11.3 |
| Exchangeable Potassium | mg·kg^-1^ | 69 | 30 | 18 | 58 |
| Available Phosphorus | mg·kg^-1^ | 42 | 12 | 89 | 7 |
| Total Nitrogen | g·kg^-1^ | 0.94 | 1.14 | 0.82 | 1.05 |

SL17, sandy-loam soil, harvested in 2017; LS17, loam-silty soil, harvested in 2017; SL18, sandy-loam soil, harvested in 2018; LS18, loam-silty soil, harvested in 2018. The soils were sampled at a depth of 0-30 cm, using Eijkelkamp cylindrical augers, at the beginning of March, just before the N fertilization at tillering, GS (growth stage) 23, according to the BBCH scale (Zadoks et al., 1974).

**Table S3.** Monthly cumulative rainfall and growing degree days (GDDs)^a^ from sowing (November) to the end of the ripening stage (June) measured in the experimental areas.

| **Year** | **Month** | **Rainfall (mm)** | | **GDDs (Σ °C∙d^-1^)** | |
| --- | --- | --- | --- | --- | --- |
|  |  | **Cigliano**  **(SL)** | **Carmagnola (LS)** | **Cigliano**  **(SL)** | **Carmagnola**  **(LS)** |
| 2016-17 | November | 158 | 257 | 238 | 250 |
|  | December | 45 | 77 | 144 | 159 |
|  | January | 4 | 12 | 97 | 111 |
|  | February | 45 | 62 | 152 | 175 |
|  | March | 69 | 69 | 349 | 356 |
|  | April | 34 | 51 | 415 | 412 |
|  | May | 79 | 77 | 554 | 558 |
|  | June | 149 | 103 | 673 | 698 |
|  | November – June | 583 | 708 | 2622 | 2719 |
|  | April – May | 113 | 128 | 969 | 970 |
| 2017-18 | November | 48 | 66 | 224 | 220 |
|  | December | 33 | 27 | 113 | 82 |
|  | January | 107 | 117 | 178 | 141 |
|  | February | 60 | 86 | 113 | 89 |
|  | March | 109 | 103 | 223 | 209 |
|  | April | 93 | 116 | 456 | 444 |
|  | May | 138 | 310 | 583 | 565 |
|  | June | 35 | 14 | 665 | 672 |
|  | November – June | 623 | 839 | 2555 | 2422 |
|  | April – May | 231 | 426 | 1039 | 1009 |

^a^GDDs: accumulated growing degree days for each month using a 0 °C base; SL, sandy-loam soil; LS, loam-silty soil. Data obtained from the Regione Piemonte agrometeorological service.

**Table S4.** The timing of the main agronomic management practices applied to the plots in the field experiments conducted in the 2016-18 period in north-west Italy.

| **Crop tecniques** | **Growth stage** | **2016-17** | | **2017-18** | |
| --- | --- | --- | --- | --- | --- |
|  |  | **Cigliano** | **Carmagnola** | **Cigliano** | **Carmagnola** |
|  |  | **SL17** | **LS17** | **SL18** | **LS18** |
| Sowing date | - | 4 November 2016 | 27 October 2016 | 31 October 2017 | 31 October 2017 |
| N fertilization | Tillering (GS 23) | 7 March 2017 | 9 March 2017 | 12 March 2018 | 14 March 2018 |
|  | Stem elongation (GS 32) | 3 April 2017 | 07 April 2017 | 18 April 2018 | 19 April 2018 |
| Harvest date | - | 05 July 2017 | 14 July 2017 | 04 July 2018 | 16 July 2018 |

SL17, sandy-loam soil, harvested in 2017; LS17, loam-silty soil, harvested in 2017; SL18, sandy-loam soil, harvested in 2018; LS18, loam-silty soil, harvested in 2018.


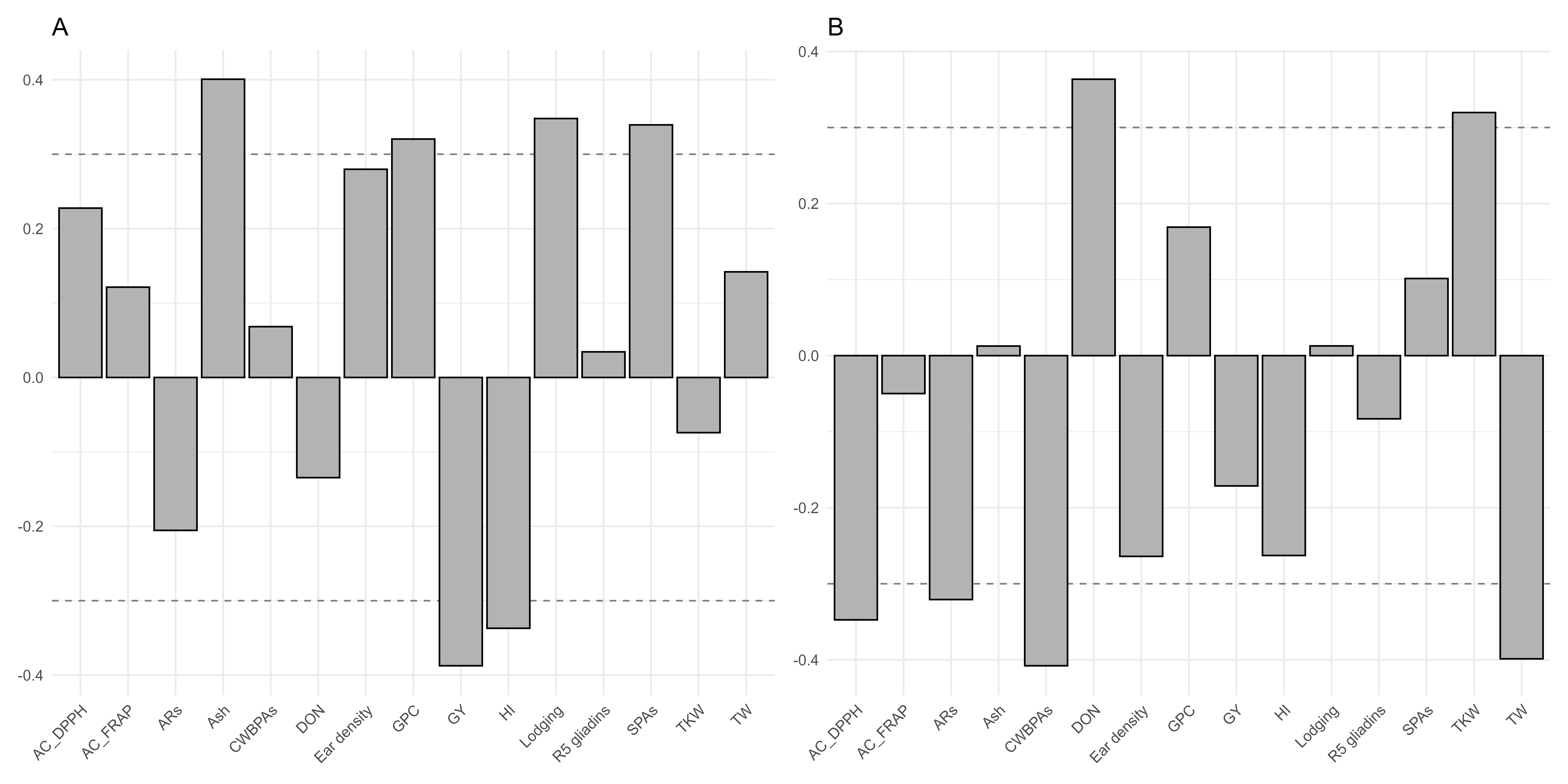
**Figure S1**. Loadings on Component 1 (A) and on Component 2 (B) of the principal component analysis reported in Figure 6.

The dotted lines delineate loadings greater than 0.3. AC, antioxidant capacity (DPPH and FRAP assays); ARs, 5‑*n*‑alkylresorcinols; CWBPAs, cell wall-bound phenolic acids; DON, deoxynivalenol; GPC, grain protein content; GY, grain yield; HI, harvest index; SPAs, soluble phenolic acids; TKW, thousand kernel weight; TW, test weight.
